# Boosting-Based Machine Learning Applications in Polymer Science: A Review

**DOI:** 10.3390/polym17040499

**Published:** 2025-02-14

**Authors:** Ivan Malashin, Vadim Tynchenko, Andrei Gantimurov, Vladimir Nelyub, Aleksei Borodulin

**Affiliations:** 1 Artificial Intelligence Technology Scientific and Education Center, Bauman Moscow State Technical University, 105005 Moscow, Russia; 2 Scientific Department, Far Eastern Federal University, 690922 Vladivostok, Russia

**Keywords:** machine learning, boosting methods, AdaBoost, Gradient Boosting, XGBoost, CatBoost, LightGBM, polymer science

## Abstract

The increasing complexity of polymer systems in both experimental and computational studies has led to an expanding interest in machine learning (ML) methods to aid in data analysis, material design, and predictive modeling. Among the various ML approaches, boosting methods, including AdaBoost, Gradient Boosting, XGBoost, CatBoost and LightGBM, have emerged as powerful tools for tackling high-dimensional and complex problems in polymer science. This paper provides an overview of the applications of boosting methods in polymer science, highlighting their contributions to areas such as structure–property relationships, polymer synthesis, performance prediction, and material characterization. By examining recent case studies on the applications of boosting techniques in polymer science, this review aims to highlight their potential for advancing the design, characterization, and optimization of polymer materials.

## 1. Introduction

Polymeric materials are widely used across various industries, including medicine [1,2,3], automotive engineering [4,5,6], packaging [7,8], and electronics [9,10,11], due to their diverse properties and adaptability. Understanding and optimizing polymer properties, as well as their manufacturing processes, is a key area of research. However, the inherent complexity of polymers, stemming from their structure–property relationships and diverse formulations, poses significant challenges for accurate modeling and prediction.

Machine learning (ML) techniques have emerged as valuable tools for addressing such challenges, with boosting algorithms being particularly effective. Methods like AdaBoost [12,13], Gradient Boosting [14,15], LightGBM [16,17], CatBoost [18,19], and XGBoost [20,21] have been used to analyze large and complex datasets, offering robust predictive capabilities and the ability to model non-linear relationships. In the context of polymer science, these algorithms have been applied to tasks such as predicting material properties, optimizing processing parameters, and designing polymer formulations.

**Figure 1:**
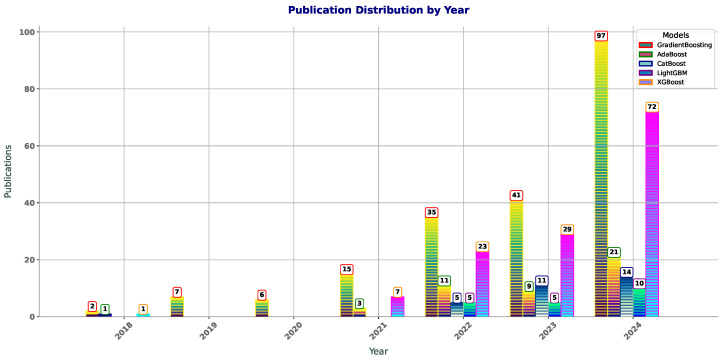
Year-wise distribution of publications featuring ‘AdaBoost’, ‘Gradient Boosting’, ‘XGBoost’, ‘CatBoost’, ‘LightGBM’, and ‘polymers’ in title or abstract.

Figure 1 shows the publication trends for boosting methods in polymer sciense —GradientBoosting, AdaBoost, CatBoost, LightGBM, and XGBoost—show shifts in popularity from 2018 to 2024. GradientBoosting has seen steady growth, from two papers in 2018 to 97 in 2024, reflecting its increasing use in polymer and other fields. AdaBoost, starting with one paper in 2018, had minimal growth, with a slight rise in 2021 and 2022, indicating limited application in polymer research. CatBoost has gained traction, especially in 2022 (5 papers) and 2023 (11 papers), due to its effectiveness with categorical data. LightGBM shows gradual growth, with a rise from zero papers in 2018–2020 to 10 in 2024, likely due to its scalability with large datasets. XGBoost grew steadily, from one paper in 2018 to 72 in 2024, driven by its versatility and strong predictive performance. In summary, GradientBoosting and XGBoost dominate, with increasing adoption, while AdaBoost and LightGBM show slower growth, and CatBoost is gaining popularity. These trends highlight the growing use of boosting methods in polymer research to improve prediction accuracy and handle complex data relationships.

**Figure 2:**
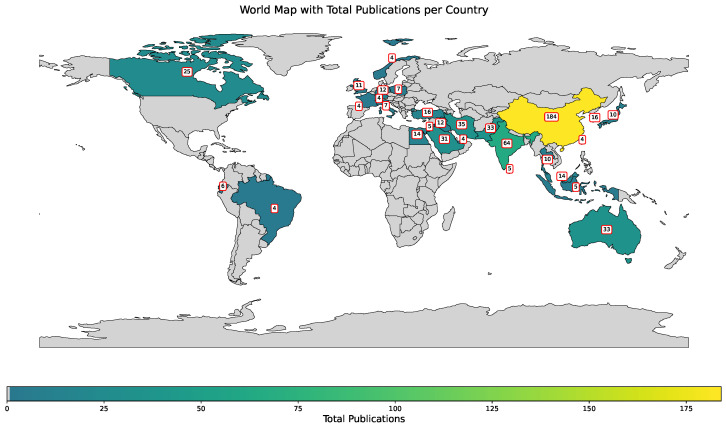
Country-wise distribution of publications featuring ‘AdaBoost’, ‘Gradient Boosting’, ‘XGBoost’, ‘CatBoost’, ‘LightGBM’, and ‘polymers’ in title or abstract.

Figure 2 shows the map with distribution of publications utilizing boosting methods in polymer research across the world. According to the provided data, countries like China (184 publications), India (64 publications), and Iran (35 publications) lead in the volume of research. Other contributors include Australia (33 publications), Canada (25 publications), and Pakistan (33 publications). Smaller but notable contributions come from countries such as the United Kingdom, France, and Turkey, each ranging between 10 to 16 publications. This distribution reflects a global interest in leveraging boosting methods for polymers, with a concentration of research efforts in leading industrial and academic hubs.

**Figure 3:**
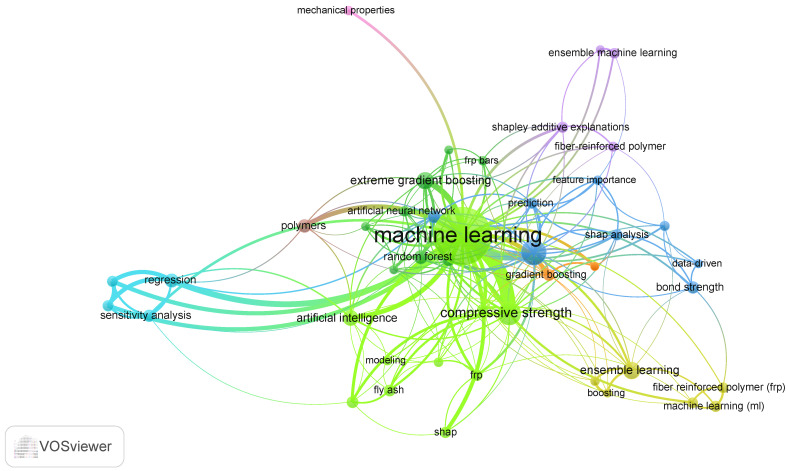
Keyword co-occurrence map based on VOSviewer analysis.

The Keywords Occurrence Map (Figure 3) highlights the integration of boosting methods, into polymer research. Techniques like gradient boosting, CatBoost, AdaBoost, and XGBoost are widely applied to predict and optimize material properties such as CS, bond strength, and mechanical performance. These methods excel in capturing complex, non-linear data relationships, enhancing accuracy and reliability. Applications include sustainability-focused studies (e.g., geopolymers, fly ash, and asphalt binders), advanced manufacturing (e.g., additive manufacturing and 3D-printing), and structural materials (e.g., FRP and ultra-high-performance concrete). Boosting models are also leveraged alongside interpretability tools like SHAP analysis and sensitivity analysis to improve understanding of key factors in photodegradation, corrosion, and microplastics.

This review aims to analyze existing research on the application of boosting methods in polymer-related tasks. The focus is on several key areas, including the prediction of mechanical, thermal, and chemical properties of polymers, the optimization of manufacturing processes and polymer blend compositions, and the integration of boosting methods with other ML approaches to improve model accuracy and interpretability.

To ensure a systematic and comprehensive review, the PRISMA [22] (Preferred Reporting Items for Systematic Reviews and Meta-Analyses) methodology was employed for identifying, screening, and selecting relevant studies. The search across multiple databases yielded a significant number of records, which were then deduplicated, screened based on titles and abstracts, and assessed for full-text eligibility. After the final selection, studies were categorized into five thematic groups, each chosen based on specific research trends and their relevance to boosting-based ML applications in polymer science.

The first category, concrete and geopolymer composites, was selected due to the increasing interest in using boosting methods to predict and optimize the properties of concrete and geopolymer composites, which play a crucial role in modern construction materials. The second category, FRP and reinforced concrete systems, includes studies focused on FRP composites and reinforced concrete structures, where ML models contribute to improving material performance, durability, and structural behavior.

The third category, material properties prediction, encompasses studies dedicated to predicting the physical, chemical, and mechanical properties of polymer-based materials, which is a key aspect of optimizing material design and applications. The fourth category, advanced manufacturing and processing, highlights research on innovative manufacturing techniques and processing methods where boosting algorithms enhance efficiency, process optimization, and defect detection. Finally, the sustainability, environmental, and structural performance category includes studies assessing the environmental impact, sustainability, and structural efficiency of polymer-based materials, addressing critical challenges related to material life cycle assessment and eco-friendly alternatives.

**Figure 4:**
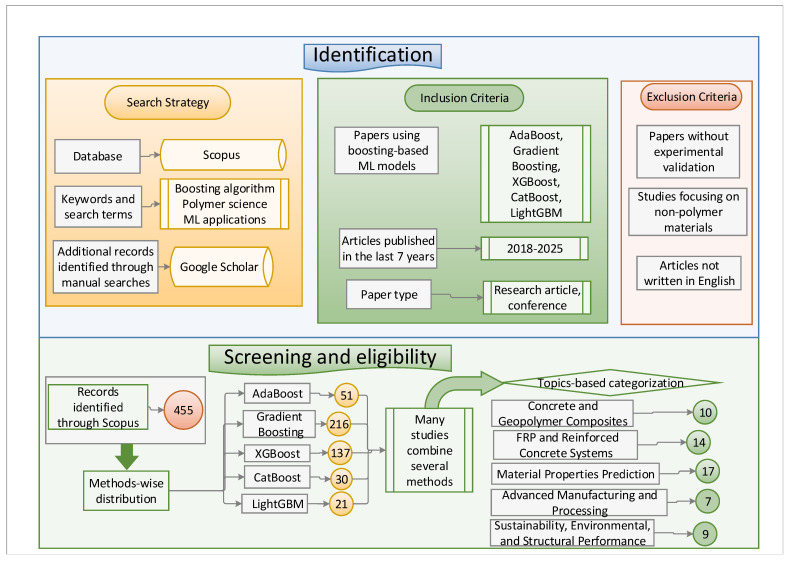
PRISMA flowchart outlining the study selection process for the systematic review.

Figure 4 is a PRISMA flowchart illustrating the study selection process for this systematic review. The diagram outlines the identification, screening, and eligibility assessment stages, leading to the final inclusion of studies.

The objective is to organize the current body of work, examine the achievements and limitations of boosting methods in this field, and suggest directions for future research. This analysis underscores the growing role of ML in materials science and explores the potential of these technologies to drive innovation in the development of advanced polymeric materials.

## 2. Theoretical Background of Boosting Methods

### 2.1. Gradient Boosting (GB)

Gradient Boosting (GB) is an ensemble method that builds a strong predictive model by combining several weak models (often decision trees) [23]. Each subsequent model is trained to predict the residual errors made by the previous models. This method can be mathematically described as follows:

Let $f_{0}\left(x\right)$ be the initial model. Typically, $f_{0}\left(x\right)$ is the constant value that minimizes the loss function. In the case of regression, this is often the mean of the target variable, ${\hat{y}}_{i}$, over all data points:

$$f_{0}\left(x\right)=\arg\min_{\gamma }\sum_{i=1}^{N}{\left(y_{i}-\gamma \right)}^{2}$$

where $\gamma =\frac{1}{N}\sum_{i=1}^{N}y_{i}$ is the mean of the target values.

At the *t*-th iteration, the model $f_{t}\left(x\right)$ is constructed by adding a new tree $h_{t}\left(x\right)$ to the previous model $f_{t-1}\left(x\right)$ in such a way that the loss function is minimized:

$$L_{t}=\sum_{i=1}^{N}{\left[y_{i}-f_{t-1}\left(x_{i}\right)-\eta h_{t}\left(x_{i}\right)\right]}^{2}$$

where:

- $y_{i}$ is the actual target value,
- $f_{t-1}\left(x_{i}\right)$ is the predicted value from the previous model,
- $h_{t}\left(x_{i}\right)$ is the new decision tree that is fitted to the residuals (errors) from $f_{t-1}\left(x_{i}\right)$,
- η is the learning rate, which controls the contribution of each tree.

The new model is then updated iteratively by adding each new tree’s contribution [24]:

$$f\left(x\right)=f_{0}\left(x\right)+\sum_{t=1}^{T}\eta h_{t}\left(x\right)$$

where *T* is the total number of iterations (trees).

In GB, the model is trained by optimizing the following objective function:

$$L\left(\theta \right)=\sum_{i=1}^{N}\ell \left(y_{i},f\left(x_{i}\right)\right)+\sum_{t=1}^{T}\Omega \left(h_{t}\right)$$

where:

- $\ell \left(y_{i},f\left(x_{i}\right)\right)$ is the loss function that measures the error of the model’s prediction,
- $\Omega (h_{t})$ is the regularization term that penalizes overly complex trees, often given by:

$$\Omega \left(h_{t}\right)=\gamma T+\frac{1}{2}\lambda \sum_{j=1}^{T}{\left(w_{j}\right)}^{2}$$

where *T* is the number of leaves in the tree, $w_{j}$ are the leaf weights, and γ and λ are regularization parameters.

The final model f(x) is an additive combination of all trees, which can be written as:

$$f\left(x\right)=f_{0}\left(x\right)+\sum_{t=1}^{T}\eta h_{t}\left(x\right)$$

This additive model helps correct the errors made by the previous trees.

To minimize the loss function, the gradient is computed of the loss with respect to the current prediction $f_{t-1}\left(x_{i}\right)$ and a new tree $h_{t}\left(x\right)$ fitted to the residuals [25]:

$$g_{i}=\nabla_{f_{t-1}\left(x_{i}\right)}L\left(y_{i},f_{t-1}\left(x_{i}\right)\right)$$

This gradient $g_{i}$ represents the residuals (the errors) from the current model. The next tree $h_{t}\left(x\right)$ is then trained to fit these residuals.

The process is similar to performing a gradient descent optimization, where each subsequent tree steps in the direction of minimizing the loss.

GB can be applied to various polymer research problems, such as predicting polymer material properties (strength, viscosity, thermal stability, etc.) based on experimental data. For instance:

$${\hat{y}}_{i}=f\left(x_{i}\right)=f_{0}\left(x_{i}\right)+\sum_{t=1}^{T}\eta h_{t}\left(x_{i}\right)$$

where $x_{i}$ represents the chemical composition and structural features of the polymer, and ${\hat{y}}_{i}$ is the predicted property (e.g., tensile strength).

GB is useful when the relationship between the polymer features and the material properties is non-linear and complex, which is often the case in polymer science [26]. By iteratively fitting trees to the residuals of previous models, GB can provide highly accurate predictions even for complex datasets. For instance Park et al. [27] proposed a boosting-based probabilistic model (NGBoost) [28] to predict the physical properties of polypropylene composites, addressing data imbalance and uncertainty.

### 2.2. AdaBoost

AdaBoost (Adaptive Boosting) is a popular ensemble learning technique that combines multiple weak classifiers to form a strong classifier [29,30]. It operates by focusing on the instances that previous classifiers misclassified, increasing their weight to make the next classifier pay more attention to those examples.

The AdaBoost algorithm works by iteratively adding weak classifiers to the ensemble, with each classifier trained on the weighted data. The general process is as follows:

The first classifier $f_{1}\left(x\right)$ is trained by minimizing the weighted loss function. The initial weight for each data point is the same, and the loss function typically used is the exponential loss function:

$$L\left(f_{1}\right)=\sum_{i=1}^{N}w_{i}{\left(y_{i}-f_{1}\left(x_{i}\right)\right)}^{2}$$

where:

- $f_{1}\left(x\right)$ is the weak classifier,
- $w_{i}$ is the weight of the *i*-th instance,
- $y_{i}$ is the true label for the *i*-th instance.

The goal is to minimize this weighted loss function to obtain the first weak classifier $f_{1}\left(x\right)$.

After the first classifier is trained, the weights of misclassified instances are increased, and the weights of correctly classified instances are decreased. The weight update for the *i*-th instance at the *t*-th iteration is given by:

$$w_{i}^{\left(t\right)}=w_{i}^{\left(t-1\right)}\cdot \exp\left(-\alpha_{t}y_{i}f_{t}\left(x_{i}\right)\right)$$

where:

- $\alpha_{t}$ is the weight of the *t*-th classifier, computed as:

$$\alpha_{t}=\frac{1}{2}\ln\left(\frac{1-\epsilon_{t}}{\epsilon_{t}}\right)$$

and $\epsilon_{t}$ is the weighted error rate of the *t*-th classifier:

$$\epsilon_{t}=\sum_{i=1}^{N}w_{i}^{\left(t-1\right)}\cdot ⊮\left(y_{i}\neq f_{t}\left(x_{i}\right)\right)$$

where $⊮(y_{i}\neq f_{t}\left(x_{i}\right))$ is an indicator function that takes the value 1 if $y_{i}$ is misclassified, and 0 otherwise.

The final strong classifier F(x) is the weighted sum of all the weak classifiers $f_{t}\left(x\right)$. Each weak classifier $f_{t}\left(x\right)$ is weighted according to its accuracy:

$$F\left(x\right)=\sum_{t=1}^{T}\alpha_{t}f_{t}\left(x\right)$$

where:

- *T* is the total number of weak classifiers,
- $\alpha_{t}$ is the weight (coefficient) assigned to each weak classifier based on its performance.

The final classification decision is typically made by applying a sign function to the output of the ensemble:

$$\hat{y}=\mathrm{sign}\left(F(x)\right)$$

In polymer research, AdaBoost can be applied to classification tasks, such as categorizing polymers based on their chemical composition, molecular structure, or resistance to external factors like temperature or chemical exposure. The task is to classify polymers into different categories (e.g., strong vs. weak, thermoplastic vs. thermoset), based on various features extracted from experimental data.

Let us consider the task of classifying polymers based on their thermal stability:

$${\hat{y}}_{i}=\mathrm{sign}\left(\sum_{t=1}^{T}\alpha_{t}f_{t}\left(x_{i}\right)\right)$$

where ${\hat{y}}_{i}$ represents the predicted category (e.g., stable vs. unstable) for the *i*-th polymer, and $x_{i}$ represents the feature vector (e.g., chemical composition, molecular weight, etc.) for the polymer.

AdaBoost works by iteratively re-weighting the data and training weak classifiers on the weighted data, giving more importance to misclassified instances. The weak classifiers are then combined to form a final strong classifier. In the context of polymer research, AdaBoost can be used for tasks such as classifying polymers based on their physical properties, chemical characteristics, or other factors. Its ability to focus on hard-to-classify instances and improve model performance makes it a useful tool for applications with complex or imbalanced data.

### 2.3. CatBoost

CatBoost (Categorical Boosting) is a gradient boosting algorithm that specializes in handling categorical features. Unlike traditional methods that rely on one-hot encoding, CatBoost introduces an innovative approach to minimize overfitting and reduce computational complexity [31]. It builds decision trees optimally using both categorical and continuous features.

Similar to other gradient boosting methods, CatBoost iteratively adds decision trees trained on residuals [32]. It handles categorical variables through ordered target statistics, which compute target values based on previous data points rather than all at once, preventing overfitting. Let *X* be a dataset and *y* be the target variable, and $x_{i}$ represent the *i*-th observation with categorical feature $C_{j}$. The key transformation for a categorical feature is computed as follows:

$$\hat{C_{j}}=\frac{1}{|S_{j}|}\sum_{x_{i}\in S_{j}}y_{i}$$

where:

- $S_{j}$ is the set of observations corresponding to the category $C_{j}$,
- $\hat{C_{j}}$ is the average target value for the categorical feature.

This transformation is performed using the ordered target statistics, where for each data point, the target statistic is computed using previous data points only to avoid data leakage.

After transforming the categorical features, the model proceeds to build decision trees, much like standard gradient boosting. The goal of each decision tree is to minimize the residual error from the previous model:

$$L_{t}=\sum_{i=1}^{N}{\left[y_{i}-f_{t-1}\left(x_{i}\right)-\eta h_{t}\left(x_{i}\right)\right]}^{2}$$

where:

- $f_{t-1}\left(x_{i}\right)$ is the previous model’s prediction,
- η is the learning rate, and
- $h_{t}\left(x_{i}\right)$ is the decision tree model trained on the residuals.

Each tree aims to minimize this residual error, adjusting the contribution of each tree using the gradient descent approach.

Once the trees are built, the final model f(x) is a weighted sum of all decision trees, where the weight of each tree is controlled by the learning rate η:

$$f\left(x\right)=f_{0}\left(x\right)+\sum_{t=1}^{T}\eta h_{t}\left(x\right)$$

where $f_{0}\left(x\right)$ is the initial model, typically, the mean of the target values.

To optimize the CatBoost model, the objective function is similar to that used in other gradient boosting algorithms. The loss function for regression, for example, is the mean squared error (MSE):

$$L\left(\theta \right)=\sum_{i=1}^{N}{\left(y_{i}-f\left(x_{i}\right)\right)}^{2}$$

The gradient of this loss function is computed to guide the model’s optimization. At each iteration, the new tree $h_{t}\left(x\right)$ is trained to fit the negative gradient of the loss function, which corresponds to the residuals from the previous model.

CatBoost also includes regularization techniques to prevent overfitting. Specifically, it uses feature combinations and permutation-driven techniques that improve model generalization by considering various ways to combine the features. The regularization term can be written as:

$$\Omega \left(h_{t}\right)=\gamma T+\frac{1}{2}\lambda \sum_{j=1}^{T}{\left(w_{j}\right)}^{2}$$

where:

- *T* is the number of leaves in the tree,
- $w_{j}$ is the weight of each leaf node,
- γ and λ are regularization parameters that control the complexity of the trees.

In polymer research, CatBoost is highly beneficial when dealing with complex datasets that contain a mix of categorical and continuous variables. For example, in the study of polymer materials, the polymerization process conditions (e.g., temperature, pressure, catalyst types) can be categorical, while properties such as the tensile strength, viscosity, or melting temperature are continuous. CatBoost can efficiently handle these mixed data types, building robust predictive models without the need for extensive pre-processing or feature engineering.

For instance, in predicting the tensile strength of a polymer based on various processing conditions and chemical additives:

$${\hat{y}}_{i}=f\left(x_{i}\right)=f_{0}\left(x_{i}\right)+\sum_{t=1}^{T}\eta h_{t}\left(x_{i}\right)$$

where $x_{i}$ includes both continuous features (such as temperature) and categorical features (such as the type of chemical additive), and ${\hat{y}}_{i}$ is the predicted tensile strength.

Since CatBoost handles categorical features directly, without the need for one-hot encoding, it reduces the computational complexity and improves both the speed and accuracy of the model, making it ideal for polymer science tasks that involve large and complex datasets.

### 2.4. LightGBM

LightGBM (Light Gradient Boosting Machine) is a gradient boosting framework that uses a histogram-based algorithm to improve both the training speed and memory efficiency [33]. Instead of using all possible feature values, LightGBM groups them into bins, which reduces the complexity of decision trees and speeds up computations. This makes LightGBM especially suitable for handling large datasets, where scalability is a concern.

The basic idea behind LightGBM is to discretize continuous features into bins. The model then makes decisions based on these bins rather than individual feature values, speeding up both the training and prediction processes. The following steps outline the key mathematical aspects of LightGBM.

For each continuous feature $x_{i}$, LightGBM groups values into bins. The binning process is as follows:

$$\mathrm{Bin}\left(x_{i}\right)=\mathrm{floor}\left(\frac{x_{i}}{\delta }\right)$$

where:

- $x_{i}$ is the value of the continuous feature,
- δ is the binning step size (which determines the size of the bins), and
- $\mathrm{Bin}(x_{i})$ represents the bin index that the value $x_{i}$ falls into.

This transformation reduces the number of unique feature values, making the model more computationally efficient.

The algorithm proceeds by building decision trees based on the binned features. The decision tree is built using a gradient-based approach, where each split is made to minimize the residual error from the previous tree. The residual error at the *t*-th iteration is:

$$L_{t}=\sum_{i=1}^{N}{\left(y_{i}-f_{t-1}\left(x_{i}\right)-\eta h_{t}\left(x_{i}\right)\right)}^{2}$$

where:

- $f_{t-1}\left(x_{i}\right)$ is the prediction from the previous model,
- η is the learning rate,
- $h_{t}\left(x_{i}\right)$ is the prediction of the new decision tree at the *t*-th step.

The tree is grown by iterating over the binned feature values and selecting the best split based on the gradient of the loss function. In LightGBM, this is performed using the histogram-based approach, which selects the optimal bin split that minimizes the loss.

In a standard decision tree, splits are found by considering all feature values. LightGBM, however, uses a histogram-based approach. Let the histogram $H_{j}$ for feature *j* be defined as:

$$H_{j}=\left\{h_{1},h_{2},\ldots ,h_{k}\right\}$$

where $h_{k}$ is the count of instances falling into bin *k* for feature *j*. The algorithm then calculates the gradient of the loss function for each bin and chooses the best split based on these gradients.

For a given candidate split on feature *j*, the gain from the split can be computed as:

$$\mathrm{Gain}=\frac{1}{N}\sum_{i\in L}{\left(\frac{\partial L}{\partial f}\right)}^{2}+\sum_{i\in R}{\left(\frac{\partial L}{\partial f}\right)}^{2}$$

where:

- *N* is the total number of instances,
- *L* and *R* represent the left and right child nodes after the split,
- $\frac{\partial L}{\partial f}$ is the gradient of the loss with respect to the feature values.

The optimal split is chosen based on the highest gain, which corresponds to the best reduction in the residual error after the split.

To prevent overfitting, LightGBM applies regularization techniques such as L2 regularization and leaf-wise pruning. The regularization term for the decision tree can be written as:

$$\Omega \left(h_{t}\right)=\gamma T+\lambda \sum_{i=1}^{T}{\left(w_{j}\right)}^{2}$$

where:

- *T* is the number of leaves in the tree,
- $w_{j}$ is the weight of the *j*-th leaf node,
- γ and λ are regularization parameters.

The regularization helps to control the complexity of the trees and ensures that the model generalizes well to unseen data.

The final model in LightGBM is a weighted sum of all the decision trees built during the boosting process:

$$f\left(x\right)=f_{0}\left(x\right)+\sum_{t=1}^{T}\eta h_{t}\left(x\right)$$

where:

- $f_{0}\left(x\right)$ is the initial model, typically the mean of the target values,
- η is the learning rate,
- $h_{t}\left(x\right)$ is the *t*-th decision tree model.

LightGBM’s ability to efficiently handle large datasets makes it useful for predicting polymer properties when scalability is a concern. In polymer science, the data used for prediction often come from a variety of sources, including experimental measurements of physical properties (e.g., tensile strength, elasticity, viscosity) and process conditions (e.g., temperature, pressure, and type of catalyst).

For instance, LightGBM can be applied to predict the thermal conductivity of a polymer based on a variety of experimental factors. The feature set $x_{i}$ might include both continuous features (e.g., temperature) and categorical features (e.g., type of polymer or catalyst), which can be binned and used to create an efficient model.

The final prediction for the polymer property ${\hat{y}}_{i}$ is given by:

$${\hat{y}}_{i}=f\left(x_{i}\right)=f_{0}\left(x_{i}\right)+\sum_{t=1}^{T}\eta h_{t}\left(x_{i}\right)$$

where $x_{i}$ is the feature vector containing both categorical and continuous features, and ${\hat{y}}_{i}$ is the predicted polymer property.

### 2.5. XGBoost

XGBoost (XGBoost) is an optimized version of the gradient boosting algorithm, designed to improve training efficiency, prediction accuracy, and model interpretability. It incorporates regularization techniques to prevent overfitting, handles sparse data, and uses more efficient training methods, such as parallelization and hardware optimizations [34].

XGBoost builds models by sequentially adding decision trees, where each tree is fitted to the residuals (errors) from the previous model. It aims to minimize the loss function with an additional regularization term to prevent overfitting.

At the *t*-th iteration, the model is updated as follows:

$$L_{t}=\sum_{i=1}^{N}{\left(y_{i}-f_{t-1}\left(x_{i}\right)-\eta h_{t}\left(x_{i}\right)\right)}^{2}$$

where:

- $y_{i}$ is the true label for instance *i*,
- $f_{t-1}\left(x_{i}\right)$ is the model’s prediction for instance *i* at the previous step,
- $h_{t}\left(x_{i}\right)$ is the prediction of the new decision tree at the *t*-th step,
- η is the learning rate.

This formulation minimizes the residual error from the previous model, and the final model prediction is the sum of all tree predictions.

XGBoost introduces regularization to penalize complexity and reduce overfitting [35]. The regularization term Ω(f) is added to the objective function to control the complexity of the model:

$$\Omega \left(f\right)=\gamma T+\frac{1}{2}\lambda \sum_{j=1}^{T}{\left(w_{j}\right)}^{2}$$

where:

- *T* is the number of leaves in the tree,
- $w_{j}$ is the weight of the *j*-th leaf,
- γ is a regularization parameter controlling the number of leaves in the tree,
- λ is a regularization parameter controlling the size of the weights.

The regularization term discourages overly complex trees with many leaves and large weights, which helps prevent overfitting [36].

The final objective function to be minimized in XGBoost combines the loss function (residuals) and the regularization term:

$$L\left(f\right)=\sum_{i=1}^{N}L\left(y_{i},f\left(x_{i}\right)\right)+\Omega \left(f\right)$$

where:

- $L(y_{i},f\left(x_{i}\right))$ is the loss function that measures the difference between the true label $y_{i}$ and the predicted value $f(x_{i})$,
- Ω(f) is the regularization term as defined earlier.

The goal of XGBoost is to find the function f(x) that minimizes this objective.

XGBoost builds trees using a greedy algorithm, which chooses the best split at each node based on the objective function. The best split *q* for a given node can be found by maximizing the gain:

$$\mathrm{Gain}\left(q\right)=\frac{1}{2}\left(\frac{{\left(\sum_{i\in L}g_{i}\right)}^{2}}{h_{L}+\lambda }+\frac{{\left(\sum_{i\in R}g_{i}\right)}^{2}}{h_{R}+\lambda }-\frac{{\left(\sum_{i\in S}g_{i}\right)}^{2}}{h_{S}+\lambda }\right)$$

where:

- $g_{i}$ and $h_{i}$ are the gradient and Hessian of the loss function (first and second derivatives),
- *L*, *R*, and *S* are the left, right, and split node, respectively.

The gain represents the reduction in the loss after the split, and the optimal split maximizes this gain [37].

Once the trees are constructed, the final prediction is obtained by summing the predictions of all trees:

$$f\left(x\right)=f_{0}\left(x\right)+\sum_{t=1}^{T}\eta h_{t}\left(x\right)$$

where:

- $f_{0}\left(x\right)$ is the initial model (often the mean value of the target),
- η is the learning rate,
- $h_{t}\left(x\right)$ is the prediction from the *t*-th tree.

The prediction for a new instance is computed by summing the predictions of all trees, weighted by the learning rate [38]. XGBoost also incorporates early stopping to prevent overfitting [39]. During training, the model evaluates performance on a validation set after each boosting round. If the performance on the validation set does not improve for a specified number of rounds (called the early stopping round), the training stops.

XGBoost is useful for predicting polymer properties when both numerical and categorical features are present. In polymer research, there are often large datasets consisting of experimental measurements (e.g., tensile strength, viscosity, thermal properties) as well as categorical features (e.g., type of polymer, catalyst used, reaction conditions). XGBoost’s ability to handle mixed types of data (numerical and categorical) and its efficient training process make it ideal for these types of predictions. For example, Ueki et al. [40] employed ML to predict grafting yields in radiation-induced graft polymerization of methacrylate ester monomers onto polyethylene-coated polypropylene fabric. XGBoost demonstrated the highest prediction accuracy, identifying monomer polarizability and O_2_ NMR shift as key factors influencing grafting efficiency.

For example, XGBoost can be used to predict the stability of a polymer under various conditions, such as exposure to UV radiation or chemical reactions, based on features like molecular structure, temperature, and type of additives used.

The final prediction for polymer property ${\hat{y}}_{i}$ can be written as:

$${\hat{y}}_{i}=f\left(x_{i}\right)=f_{0}\left(x_{i}\right)+\sum_{t=1}^{T}\eta h_{t}\left(x_{i}\right)$$

where $x_{i}$ is the feature vector containing both numerical and categorical features, and ${\hat{y}}_{i}$ is the predicted polymer property.

## 3. Case Studies

### 3.1. Concrete and Geopolymer Composites

Zhao et al. [41] developed a high-generalizability ensemble ML (EML) framework to predict the homogenized mechanical properties of short FRP composites. Using a stacking algorithm, the EML combines Extra Trees (ET), XGBoost, and LightGBM as base models. The framework incorporates a micromechanical model employing a two-step homogenization algorithm verified for its accuracy in modeling composites with randomly distributed fibers, integrating finite element simulations for robust datasets. The results obtained show the EML achieves high accuracy (R^2^ values of 0.988 and 0.952 for train and test datasets) and efficient generalization on experimental data, outperforming computationally intensive high-fidelity models. SHAP analysis reveals the Young’s modulus of the matrix [42,43], fiber, and fiber content as key factors influencing the homogenized properties, with anisotropy dominated by fiber orientation. This framework reduces computational costs significantly while maintaining precision and interpretability, showcasing its applicability for advanced composite material design.

A two-level stacking algorithm framework (Stacking-CRRL) combining Catboost, RF, Ridge Regression (RR), and LASSO regression is proposed by Zhang et al. [44] for predicting the axial compression load capacity of steel-reinforced concrete columns (SRCCs) clad in CFRP. Sparse data were balanced using SMOTE, and 12 predictive features were selected after eliminating redundancy via Spearman correlation analysis. Catboost, RFR, and RR were the chosen base learners, with LASSO as the meta-learner. The results obtained indicate superior predictive performance of the Stacking-CRRL model compared to individual models, traditional ML methods, and simulation techniques. SHAP analysis further elucidated feature impacts on SRCC load capacity.

Ultra-high-performance geopolymer concrete (UHPGC) [45] offers a sustainable and economical alternative to ultra-high-performance concrete (UHPC), delivering comparable mechanical performance. Despite its potential, the absence of a robust mix design methodology limits its broader adoption. Katlav et al. [46] employed ensemble ML models, including RF, XGBoost, LightGBM, and AdaBoost, to predict the CS of UHPGC using a dataset of 181 test results with 13 input features. XGBoost emerged as the top-performing model, achieving an R^2^ of 0.948 and low error metrics. Feature importance and SHAP analyses identified age, fiber, silica fume, Na_2_SiO_3_, and water content as critical factors influencing CS. A user-friendly graphical user interface (GUI) was developed for practical CS predictions, reducing reliance on experimental tests. While promising, the model’s reliability could improve with expanded datasets and exploration of advanced AI techniques like deep learning.

Geopolymers, made from waste materials rich in aluminosilicate, present a promising alternative to traditional Portland cement. Research into GPCs is advancing, but laboratory testing remains time-intensive and costly. ML offers a faster, cost-effective method to predict the CS of these materials. Wang et al. [47] utilized a decision tree (DT) model and two ensemble methods—AdaBoost and Random Forest (RF)—were employed to estimate CS. Results showed that ensemble models outperformed the DT model, with R^2^ values of 0.90 for AdaBoost and RF, compared to 0.83 for DT. Additionally, lower errors, such as MAE and RMSE, confirmed the ensemble models’ higher accuracy. The findings emphasize ML’s potential in accelerating material property analysis for the construction industry.

Geopolymers, made from aluminosilicate-rich waste, are a promising alternative to traditional cement. Khan et al. [48] investigated predicting the CS of GPCs using ML techniques. Three models were used: support vector machine (SVM), GB, and XGBoost. Ensemble methods like GB and XGB outperformed SVM, with XGB achieving the highest R^2^ of 0.98. SHAP analysis revealed ground granulated blast-furnace slag (GGBS) as a significant positive factor for CS. Other factors like NaOH molarity and fly ash had mixed effects. These findings demonstrate ML’s potential to create fast, cost-effective solutions for eco-friendly construction materials.

GeoPC is a sustainable alternative to traditional concrete, offering environmental benefits and reliable strength performance. Zhou et al. [49] employed three ensemble ML models—Gradient Boosting Regressor (GBR), AdaBoost, and XGBoost to predict GPC’s CS and split-tensile strength (STS). Among these, XGBoost achieved the highest accuracy, with $R^{2}$ values exceeding 0.90 and lower error metrics, including MAE and RMSE. Sensitivity analysis revealed that blast furnace slag (BFS), curing duration (CD), and fine aggregate quantity were critical factors influencing GPC’s mechanical properties. K-fold analysis confirmed XGBoost’s superior performance compared to GB and AdaBoost. These results demonstrate the potential of ensemble ML models for precise GPC property prediction, enabling improved quality control and on-site adaptability in sustainable construction practices.

Amin et al. [50] explored the application of ML to predict the CS of geopolymer concrete (GeoPC), using a dataset of 481 mixes and nine input variables. Four ML models — SVM, multi-layer perceptron neural network (MLPNN) [51], AdaBoost regressor (AR), and RF—were compared to identify the most accurate predictor. Ensemble methods (AR and RF) outperformed individual techniques (SVM and MLPNN), with RF achieving the highest accuracy, yielding an $R^{2}$ of 0.95. Statistical analysis and k-fold evaluation validated the superior performance of RF, which exhibited lower error metrics (MAE, RMSE) and closer agreement between predicted and experimental results. Sensitivity analysis revealed curing time, curing temperature, and specimen age as the most significant factors influencing GeoPC’s CS, contributing 22.5%, 20.1%, and 18.5%, respectively. These findings highlight the efficiency of ensemble ML models in reducing experimental effort while promoting sustainable and cost-effective construction practices through enhanced GeoPC adoption.

Ansari et al. [52] aimed to predict the CS of GeoPC incorporating fly ash (FlA) using various ML models. Three models were tested: linear regression (LR), ANN, and AdaBoost. The AdaBoost model outperformed both LR and ANN, achieving the highest accuracy with a correlation coefficient (R^2^) of 0.944, root mean squared error (RMSE) of 2.506, and mean absolute error (MAE) of 1.259 in the training phase. In contrast, the LR model performed poorly with an R^2^ of 0.701 and RMSE of 5.805. The study used 154 datasets, with 70% for training and 30% for testing, and evaluated model accuracy through R^2^, RMSE, and MAE. The AdaBoost model showed the best predictive performance with minimal error and deviation from experimental results. The findings emphasize the efficiency of ensemble learning models like AdaBoost in improving the prediction of CS in GPC, saving both time and resources compared to traditional experimental methods.

Dodo et al. [53] investigated the use of supervised ML algorithms (MLAs) to predict the mechanical properties of fly ash/slag-based geopolymer concrete (FASBGeoPC) [54]. AdaBoost and Bagging with an ANN ensemble model were employed to predict CS using 156 data points, considering parameters like GGBS, alkaline activator, fly ash, SP dosage, NaOH molarity, aggregate, and temperature. Python programming in Anaconda Navigator with Spyder was used for model development and validation. Statistical evaluation, including MAE, RMSE, and R^2^, confirmed that the ensemble methods outperformed individual models, with AdaBoost-ANN achieving the highest R^2^ of 0.914. The Shapley analysis identified GGBS, NaOH molarity, and temperature as the most influential parameters in determining CS. Additionally, ensemble methods, such as boosting and bagging, demonstrated more reliable performance, with AdaBoost-ANN showing the least errors and highest accuracy compared to other models. The research indicates that these models, especially the ensemble methods, are effective for predicting FASBGPC properties and can be applied in civil engineering.

Wudil et al. [55] introduced an innovative approach combining ensemble ML and experimental data to predict the carbon dioxide footprint (CO^2^-FP) of fly ash GeoPC. Utilizing Adaboost to enhance decision tree regression (DTR) and support vector regression (SVR), the methodology accurately captures complex relationships between material features and CO^2^ emissions. Optimal feature combinations (Combo-3) yielded the best predictive performance, with Adaboost-DTR achieving the highest accuracy (CC = 0.9665, NSE = 0.9343). Evaluation through metrics like MAE and RMSE, alongside SHAP analysis, emphasized the critical role of NaOH, curing temperature, and fly ash content in emissions. While dataset limitations and applicability to broader concrete types are challenges, the findings support material optimization for sustainable construction and integration with IoT systems for real-time CO^2^ monitoring. Future work includes expanding datasets and exploring other concrete formulations to enhance generalizability.

Table 1 summarizes studies that applied ensemble boosting techniques in predicting the properties of concrete and GPCs across a variety of applications. The table includes details on the materials or properties predicted, the boosting methods used, model performance, key influencing factors, and additional techniques, such as feature importance analysis and data balancing approaches.

### 3.2. FRP and Reinforced Concrete Systems

Developments in FRP composites significantly impact civil engineering, especially in strengthening concrete structures. ML models for predicting FRP–concrete bond strength often fall short of optimal performance. Kim et al. [56] employed the CatBoost algorithm to enhance prediction accuracy, utilizing data from 855 single-lap shear tests. CatBoost outperformed other ensemble methods (XGBoost, HGBoost, RF) with metrics like lower RMSE (2.31) and higher R^2^ (0.96). It also surpassed ANN-based models, confirming its efficacy with small datasets and categorical features. This highlights CatBoost’s robustness and suitability for bond strength prediction tasks in FRP–concrete systems.

FRP have proven to be effective in strengthening reinforced concrete (RC) structures, but accurately assessing their fire resistance remains a challenge due to limited guidance in building codes. Kumarawadu et al. [57] explored the use of ML to predict the fire resistance of FRP-strengthened RC beams, using a dataset of over 21,000 data points from numerical simulations and experimental tests. Twelve ML models, including ensemble methods like XGBoost and CatBoost, were evaluated, with some achieving accuracy rates over 92%. The study also utilized Bayesian optimization for model tuning and SHAP analysis to assess the influence of key features such as loading ratio and insulation depth. The results highlighted that ensemble ML models outperformed traditional methods, showcasing their ability to accurately predict fire resistance. Key factors affecting fire resistance included the loading ratio, area of tensile reinforcement, insulation depth, and concrete cover thickness. The study concludes that ML models, especially ensemble techniques, provide valuable insights for optimizing fire safety in FRP-strengthened RC beams, with further research needed to expand the dataset to cover a wider range of real-world scenarios.

Wang et al. [58] utilized ML to identify the factors affecting the contribution of externally bonded fiber-reinforced polymer (FRP) composites to the shear strength of reinforced concrete (RC) beams. A comprehensive database of 442 FRP-strengthened RC beams was created, and anomaly detection was applied using the isolation forest algorithm. Six ML models were trained, with XGBoost achieving the highest prediction accuracy compared to traditional equations commonly used in design codes. Key influencing factors for the FRP contribution to shear strength were identified, including the effective height of FRP, shear span ratio, and reinforcement method. The trained models revealed that different reinforcement methods, such as U-wrap, full wrapping, and side-bonding, significantly affect the shear contribution of FRP. Further analysis of parameter importance showed that the effective height of FRP had the greatest impact. A new equation for predicting the shear strength contribution of FRP was derived, integrating the ML models and key influencing parameters, such as the shear span ratio and reinforcement method. This combination of ML and traditional models provides a novel, interpretable method for predicting shear strength in RC beams.

GFRP bars are prone to bonding failure due to their low bond strength with concrete. Mahmoudian et al. [59] utilized four tree-based ML models—Decision Tree, RF, AdaBoost, and XGBoost—to predict flexural bond strength and failure modes at the concrete–GFRP interface. Genetic algorithms were employed to optimize model hyperparameters, increasing R^2^ scores by up to 4%. The XGBoost classifier achieved perfect accuracy (100%) in predicting failure modes from test data. Additionally, Shapley Value analysis provided a detailed understanding of feature importance, enhancing model interpretability. These findings highlight the potential of ML in advancing GFRP–concrete interface reinforcement methods.

Mahmoudian et al. [60] evaluated the bond strength of FRP rebars in ultra-high-performance concrete (UHPC) using ML models trained on experimental datasets. The variables considered included rebar type, diameter, elastic modulus, tensile strength, concrete CS, embedment length, and test method. Various boosting ML models, including AdaBoost, CatBoost, Gradient Boosting, XGBoost, and Hist Gradient Boosting, were tested, with XGBoost achieving the highest R^2^ score of 0.95 and the lowest RMSE of 2.21. Shapley values analysis identified tensile strength, elastic modulus, and embedment length as the most influential factors. Hyperparameter tuning significantly improved model accuracy, with ensemble approaches like Voting Regressor further enhancing prediction reliability. The study also highlighted the advantages of ML models over traditional methods, which often lack adaptability across diverse scenarios. Additionally, a user interface was developed to facilitate the practical application of these models in structural engineering, providing a customizable platform for engineers to predict bond strength in FRP-reinforced UHPC.

Wang et al. [61] introduced a genetic evolutionary deep learning framework to assess the fire resistance of FRP-strengthened reinforced concrete (RC) beams. The approach uses the Light Gradient-Boosting Machine (LightGBM) algorithm, optimized with a Genetic Algorithm, and Genetic Programming (GP) to predict fire resistance performance. A dataset of 20,000 data points from numerical models and experimental studies was used. The LightGBM model achieved high accuracy, with R^2^ values of 0.923 for fire resistance time and 0.789 for deflection at failure, while the GP model provided explicit equations but with lower accuracy (R^2^ values of 0.642 and 0.643). The study identified that geometric features, such as insulation thickness and reinforcement area, have a significant impact on fire resistance, which traditional models fail to capture. A graphical user interface (GUI) was developed, enabling engineers to use these insights without coding skills. Additionally, model interpretability techniques like SHAP values and trend analysis were employed to enhance the practical application of the model in engineering decisions.

Hu et al. [62] presented an ML-assisted framework for optimizing the stacking sequence and orientation of CFRP/metal composite laminates, aiming to enhance mechanical properties under quasi-static loading. By integrating experimental data with finite element simulations, the study expands ML analysis in composite material design. Nine ML models, including XGBoost and gradient boosting, were evaluated for their ability to predict tensile and bending strengths. XGBoost and gradient boosting excelled in tensile strength predictions, while decision trees, KNN, and RF performed best in bending strength predictions. The study identifies optimal layup sequences, with sequence 2 showing superior mechanical properties. The combination of ML, numerical, and experimental approaches provided deep insights into CFRP/metal composites’ performance. Overall, the findings offer valuable design references and highlight the importance of advanced analytical models for composite material optimization.

Aydın et al. [63] examined the wear behavior of multiwall carbon nanotube (MWCNT)-doped non-crimp fabric carbon fiber-reinforced polymer (NCF-CFRP) composites. The results showed that a 1 wt% MWCNT reinforcement reduced wear loss by 48.1% and 61.1% under 10 N and 30 N loads, respectively, over a sliding distance of 1000 m. Various ML models were evaluated for predicting wear loss, including Deep Multi-Layer Perceptron (DMLP), RF Regression (RFR), Gradient Boosting Regression (GBR), linear regression (LR), and polynomial regression (PR). Among these, the DMLP model exhibited the best predictive performance, achieving an R^2^ of 0.9726 in testing, and showed effective generalization without overfitting across varying loads. The study also found that maximum wear resistance occurred at 1 wt% MWCNT content, with wear loss increasing as load and sliding distance grew. SEM and EDS analyses revealed matrix delamination and CF fractures at higher loads. The study is the first to use ML to predict the wear behavior of epoxy matrix hybrid nanocomposites.

Li et al. [64] proposed an ML-based method using RF and AdaBoost algorithms to predict the bond strength between basalt fiber-reinforced polymer (BFRP) bars and concrete in corrosive environments. The model was trained on 355 samples, incorporating factors such as corrosion, concrete strength, and BFRP bar properties. The AdaBoost model outperformed RF, achieving an R^2^ value of 0.925, RMSE of 0.0769, and MAE of 0.0589, showing high accuracy. The SHAP method was used to analyze the impact of various factors on bond strength, with the corrosion factor being the most influential. The ML models outperformed traditional empirical models, which had a much higher coefficient of variation. This research highlights the potential of ML techniques in predicting bond strength, offering a more reliable and generalizable alternative to traditional methods. These models can be extended to other types of FRP concrete systems, enhancing prediction accuracy in the field.

Khodadadi et al. [65] introduced a novel Particle Swarm Optimization [66,67]-Categorical Boosting (PSO-CatBoost) model for predicting the CS of CFPR Confined-Concrete (CFRP-CC). The model, trained on 916 experimental results from 105 studies (1991–2023), integrates PSO with CatBoost, leveraging advanced feature evaluation methods, such as SHAP and Permutation Feature Importance (PFI). Comparative analysis shows the proposed model achieved the highest R^2^ (0.9572 for testing), and lowest MSE, MAE, and RMSE, outperforming six other ML algorithms and traditional empirical models. Key influencing factors identified include the CFRP reinforcement ratio and unconfined concrete CS. The PSO-CatBoost model represents a significant advancement, providing higher predictive accuracy and generalizability. A user-friendly graphical interface further enhances its practical applicability, setting a new standard for predictive modeling in CFRP-CC research. This work underscores the transformative potential of data-driven approaches in engineering domains.

Accurately predicting the compressive behavior of FRP-confined concrete is critical for optimizing structural designs, meeting safety standards, and minimizing costs and environmental impacts. Alizamir et al. [68] examined four ML models—GBRT, RF, ANNMLP, and ANNRBF—using data from 765 circular specimens. GBRT improved predictions of the strength ratio ($f_{cc}^{\prime }/f_{co}^{\prime }$) with an RMSE reduction of up to 69.94% compared to empirical models. ANNMLP excelled in predicting the strain ratio ($\epsilon_{cc}/\epsilon_{co}$), outperforming GBRT, RF, and others by up to 83.74% in RMSE. These findings demonstrate that ML models, particularly GBRT and ANNMLP, outperform empirical methods, offering enhanced precision for FRP-confinement design. Future research could refine these models using advanced algorithms, robust feature selection, and extended datasets to further improve accuracy and generalization capabilities.

FRP rebars are increasingly used in construction due to infrastructure demands and the need for seawater and sea sand concrete. Amin et al. [69] sought to estimate the flexural capacity of FRP-reinforced concrete beams using decision tree (DT) and gradient boosting tree (GBT) models, incorporating six input parameters, such as beam depth and concrete CS. The models were trained on 60% of the dataset and validated on 40% using the correlation coefficient (R), mean absolute error (MAE), and root mean square error (RMSE). The GBT model outperformed DT, achieving higher R values (0.94 during validation) and a regression slope closer to 1 (0.83 for GBT vs. 0.75 for DT). Sensitivity analysis identified beam depth as the most critical factor influencing flexural strength. While the GBT model showed superior accuracy compared to prior gene expression programming (GEP) models, the American Concrete Institute (ACI) equations remain more reliable overall. Combining R with additional error indices like MAE ensures robust AI model evaluation.

Amin et al. [70] developed ML models to predict the interfacial bond strength (IBS) of FRP laminates on concrete prisms with grooves. Three ensemble models—RF regression, XGBoost, and Light Gradient Boosting Machine (LIGHT GBM)—were evaluated. The models were trained using 70% of the dataset, with the remaining 30% used for validation. LIGHT GBM outperformed the other models, achieving an R^2^ of 0.942 for the training data and 0.865 for the testing data, demonstrating its superior accuracy. A SHAPASH analysis revealed that the elastic modulus × thickness of FRP and the width of the FRP plate were the most influential factors on IBS. All models showed reliable performance, but LIGHT GBM provided the highest prediction precision, with low RMSE and MAE values. The results highlight the potential of LIGHT GBM as a robust and efficient tool for predicting IBS in FRP-retrofitted concrete structures.

Tian et al. [71] investigated the influence of FRP bar surface types on bond strength to concrete and developed a practical equation for predicting interfacial bond strength. A database of 158 pull-out test results for helically wrapped and ribbed FRP bars was compiled, considering eight influencing factors, including rib spacing (wc), rib width (wf), rib height (rh), and concrete properties. Twelve ML models were trained, with CatBoost achieving the highest accuracy, reducing RMSE by 58.3% compared to the best existing equation. Geometric indices like wf/d, wc/d, and fc were identified as the most critical factors. A new equation derived from the CatBoost model and existing formulas integrated predictive accuracy with physical interpretability. This equation offers a robust tool for practical design applications. Future research should focus on optimizing geometric indices for varying FRP bar diameters.

Table 2 summarizes studies that applied ensemble boosting techniques to predict properties of polymer-based materials, specifically in civil engineering contexts, such as FRP-concrete bond strength, shear strength of FRP-RC beams, and fire resistance of FRP-strengthened RC beams. It outlines the boosting algorithms employed, the materials and properties predicted, the datasets used, model performance metrics (such as R^2^, RMSE, and MAE), key influencing factors, and additional techniques or analyses used to improve prediction accuracy.

### 3.3. Material Properties Prediction

Cheng et al. [72] introduced an ML-based method for predicting the friction coefficient of polymer–metal pairs by analyzing friction noise across a wide temperature range (−120 °C to 25 °C) and under various working conditions. Three ML algorithms—XGBoost, LightGBM, and CatBoost—were used to establish a relationship between the time-frequency features of the friction noise and the friction coefficient. Among the models, LightGBM provided the highest accuracy for friction coefficient prediction, while XGBoost excelled in predicting aluminum alloy–polymer pairs. The results show that LightGBM achieved average RMSE and R^2^ values of 0.0135 and 0.615, respectively. The study demonstrated that ML can effectively predict the friction coefficient for different polymer–metal pairs, providing a basis for real-time, in situ monitoring of tribological properties. Future research will focus on applying this approach to a wider range of polymer materials and contact modes, and improving the algorithm’s robustness under more severe environmental conditions.

Fatriansyah et al. [73] investigated the use of Simplified Molecular Input Line Entry System (SMILES) descriptors in ML models to predict the glass transition temperature (Tg) of polymers. Five models—k-nearest neighbors (KNN), support vector regression (SVR), XGBoost, ANN, and recurrent neural network (RNN)—were applied to predict Tg. The research highlights that SMILES descriptors with fewer than 200 characters are insufficient for accurate predictions, while those over 200 characters reduce model performance due to the curse of dimensionality. The ANN model achieved the highest R^2^ of 0.79, but the XGBoost model, with an R^2^ of 0.774, showed greater stability and faster training times, making it the preferred model. The study also found that the One Hot Encoding (OHE) method outperformed Natural Language Processing (NLP) in terms of training efficiency. Validation of the XGBoost model on new polymer data showed robust performance with an average deviation of 9.76% from actual Tg values. This research underscores the need for optimizing SMILES conversion and model parameters to improve prediction accuracy, with future work aimed at enhancing model generalizability.

Ascencio-Medina et al. [74] explored the dielectric permittivity of polymers, which is influenced by electronic, ionic, and dipolar polarization mechanisms. A dataset of 86 polymers was analyzed to develop two Quantitative Structure–Property Relationship (QSPR) models using the GB. From an initial 1273 descriptors, the most relevant ones were selected using a genetic algorithm. The GBR models showed high R^2^ values of 0.938 and 0.822 for training and test datasets, respectively. An Accumulated Local Effect [75] (ALE) analysis was conducted to examine the relationship between the selected descriptors and their impact on permittivity, revealing key descriptors that positively and negatively affect dielectric properties. Compared to other ML models like multiple linear regression (MLR) [76] and partial least squares (PLS) [77], GBR models excelled in handling non-linear relationships and multicollinearity. The results highlight the potential of GBR models in accurately predicting dielectric permittivity, offering insights for the design of polymer materials with desired electrical properties. This approach can accelerate polymer development, reducing the need for extensive experimental testing.

Recently, the Ramprasad group introduced a QSPR model for predicting $E_{gap}$ values of 4209 polymers, achieving an R_2_ score of 0.90 and an RMSE of 0.44 at an 80/20 train-test split. Goh et al. [78] proposed an improved model, LGB-Stack, utilizing a two-level stacked generalization with LightGBM. Four molecular fingerprints were calculated from SMILES strings and reduced via recursive feature elimination to enhance input features for training. The model combines weak learners’ outputs to form a strong final prediction model. LGB-Stack achieved R^2^ and RMSE scores of 0.92 and 0.41 at the 80/20 split and further improved to 0.94 and 0.34 at a 95/5 split, surpassing the benchmark Ramprasad model [79]. This demonstrates LGB-Stack’s effectiveness in accurately predicting polymer properties while offering a foundation for future enhancements and potential applications in transfer learning.

Rajaee et al. [80] evaluated the efficacy of decision tree and AdaBoost algorithms in predicting the mechanical and fracture properties of polypropylene nanocomposites reinforced with nanoparticles and toughened with thermoplastic elastomers. AdaBoost outperformed decision tree models in accuracy for predicting tensile strength, Young’s modulus, elongation at break, elastic work, and plastic work. AdaBoost achieved an R^2^ value of 0.90 for Young’s modulus and demonstrated lower mean absolute percentage errors (<4% for some parameters). Sensitivity analysis identified thermoplastic polyolefin (TPO) [81] levels and nanoparticle content as the most influential features, significantly affecting tensile strength and Young’s modulus. Results showed that low TPO levels with high nanoparticle content yielded the highest mechanical strength, while increasing TPO influenced other parameters like elongation at break non-linearly. These findings underline the superiority of AdaBoost in handling complex datasets and the pivotal role of material composition in determining mechanical properties.

Abdi et al. [82] explored the effectiveness of various ML models, including CatBoost, LightGBM, XGBoost, AdaBoost, GBDT, ET, DT, and RF, to predict tetracycline (TC) photodegradation from wastewater using metal–organic frameworks [83,84] (MOFs). A dataset of 374 data points was used, with input parameters like catalyst dosage, antibiotic concentration, illumination time, solution pH, and the MOFs’ surface area and pore volume. The CatBoost model outperformed other models, achieving the highest accuracy with an AAPRE of 1.19% and an STD of 0.0431. This model accurately predicted TC degradation and followed the expected trends with varying operational parameters. Outlier detection confirmed the reliability of CatBoost, with 85% of predictions having errors below 1%. The results suggest that CatBoost is a reliable and efficient tool for predicting TC degradation in environmental applications like wastewater treatment.

Surface modification with hydrophilic polymer coatings offers a sustainable solution to prevent membrane clogging and reduce replacement frequency in water treatment systems. By combining molecular descriptors from RDKit and time-domain NMR (TD-NMR) data, Okada et al. [85] developed an ML approach for feature selection to predict surface properties. Polyacrylamide coatings were synthesized via UV-initiated copolymerization of ionic and nonionic monomers on PET films, with cross-linkers influencing the polymer chain dynamics. TD-NMR revealed differences in chain mobility linked to structural variations in cross-linkers, while contact angle measurements quantified surface hydrophilicity. Feature selection using Gradient Boosting Machine-Recursive Feature Elimination (GBM-RFE) demonstrated superior accuracy, identifying key molecular and dynamic properties influencing hydrophilicity. The findings highlight the importance of combining molecular descriptors and TD-NMR data to advance the development of hydrophilic polymer coatings and material-specific informatics methodologies.

Salehi et al. [86] investigated the use of ensemble ML (EML) models to predict the rheological properties of recycled plastic modified bitumen [87] (RPMB). Four models—RF, XGBoost, CatBoost, and Light Gradient-Boosting Machine (LightGBM)—were developed to predict complex shear modulus and phase angle under unaged and short-term aged conditions. Among these, the CatBoost model achieved the highest performance, with R^2^ values of 0.98 for complex shear modulus and 0.93 for phase angle. SHAP analysis revealed that the penetration of base bitumen and the quantity of recycled plastic, especially HDPE pellets, were crucial factors affecting these properties. The study used various techniques, such as partial dependence plots and individual conditional expectation plots, to analyze feature interactions and validate model predictions. The data-driven models offer a cost-effective and efficient alternative to traditional laboratory testing for RPMB mixtures, providing valuable insights for material and pavement engineers. Future work could enhance model accuracy by incorporating larger datasets and other plastic types.

Polypropylene composites (PPCs) [27,88] are increasingly utilized due to their versatility, with heat deflection temperature (HDT) serving as a critical property indicator. To address the lack of theoretical equations linking material composition to HDT, an ML approach was proposed by Chonghyo et al. [89]. Among three algorithms—MLR, XGBoost, and CatBoost—CatBoost emerged as the most effective model for HDT prediction, achieving the highest $R^{2}$ value (0.8965) and lowest RMSE (7.3477) for the entire dataset. When tested on a subset of 59 "same recipes," CatBoost maintained superior accuracy (R^2^ = 0.9801, RMSE = 2.6105). Its ordered encoding approach efficiently handled categorical data, outperforming mean encoding in MLR and XGBoost. A novel dimensionless number “A” was introduced to normalize and analyze variations within categorical groups, providing insights into HDT distributions. These results highlight CatBoost’s potential in optimizing PPCs by reducing experimental trial and error.

Chepurnenko et al. [90] focused on developing ML models to predict the rheological properties of polymers from experimental stress relaxation curves. The research employed metaheuristic approaches, local search and evolutionary algorithms, to solve combinatorial optimization problems, with a focus on decision tree construction. CatBoost Regressor was used to solve the regression problem, and data normalization and regularization methods were applied to improve model accuracy. The models, developed using generated datasets for the EDT-10 epoxy binder, predict rheological parameters like initial relaxation viscosity and velocity modulus. Performance evaluation showed the models achieved low errors, with the maximum MAPE error of 0.86 and minimum MSE of 0.001, validating their effectiveness. Future work will explore expanding ML tools, including k-nearest neighbors and support vector regression.

Precise control in laser-based powder bed fusion (PBF-LB) of polymers is essential for ensuring the quality of aerospace and automotive components. Hofmann et al. [91] employed ML to predict local solidity using thermal and temporal features extracted from the melt’s temperature profile, with infrared thermography data integrated with X-ray micro-computed tomography using LightGBM. Key predictors of porosity include the peak temperature of the melt and adequate reheating of subsurface layers. High prediction accuracy is achieved with a small voxel size and adjacent thermal data. The findings support detecting process defects and optimizing parameters without post-process testing. Future work includes extending datasets to new materials, geometries, and process parameters, ultimately enabling closed-loop feedback control systems to prevent defects and enhance industrial applications.

Gadagi et al. [92] investigated the use of ML techniques, specifically, Gradient Boosting Machine (GBM), AdaBoost, and XGBoost, to predict the surface roughness of jute/basalt epoxy composites in turning processes. The experiments, guided by Taguchi’s L27 array, examined the effects of spindle speed, feed rate, and depth of cut on surface roughness. Among the models, XGBoost demonstrated the highest predictive accuracy, with minimal errors in both training and testing datasets. The optimal turning parameters for achieving the minimum surface roughness of 0.773 μm were identified as 1500 RPM spindle speed, 0.05 mm/rev feed rate, and 0.3 mm depth of cut. An analysis of variance (ANOVA) highlighted that feed rate and spindle speed significantly impacted surface roughness, while the depth of cut showed minimal effect. ML insights revealed that the feed rate had the greatest influence on surface roughness, followed by the spindle speed and depth of cut. The findings emphasize XGBoost’s superior performance in predicting surface roughness and the effectiveness of ML in optimizing manufacturing processes.

Wang et al. [93] explored the hybridization of the Imperialist Competitive Algorithm (ICA) [94,95] with the Light Gradient Boosting Machine (LightGBM) to predict the CS of geo-polymer concrete (CSGCo). The hyperparameters of the LightGBM model were optimized using ICA to enhance its accuracy. The hybrid ICA-LightGBM model was compared to the traditional LightGBM and four ANN topologies, including multi-layer perceptron (MLP), radial basis function (RBF), generalized feed-forward neural network (GFFNN), and Bayesian regularized neural network (BRNN). The evaluation was based on R^2^, RMSE, and VAF metrics, with the ICA-LightGBM outperforming all other models in terms of prediction accuracy. Specifically, the ICA-LightGBM achieved an R^2^ of 0.9871 (training) and 0.9805 (testing), significantly outperforming the traditional LightGBM and ANN models. The results confirm that ICA is an effective optimizer for improving LightGBM’s predictive capabilities. This hybrid model can be used for accurate predictions of CSGCo, contributing to enhanced safety and efficiency in civil and construction applications.

Ahmad et al. [96] explored the use of ML algorithms to predict the CS of high-calcium fly-ash-based GPC. The study compared the performance of ensemble ML techniques—boosting and AdaBoost—against the individual ANN approach. The results show that boosting performed the best, achieving an R^2^ of 0.96, while AdaBoost reached 0.93, and ANN lagged behind with an R^2^ of 0.87. Boosting also had the lowest error values for MAE, MSE, and RMSE, highlighting its high prediction accuracy. Sensitivity analysis revealed that fly ash contributed significantly (45.3%) to the prediction of CS. The study concludes that ensemble techniques like boosting and AdaBoost are highly effective for predicting the mechanical properties of GPC, with boosting proving to be the most accurate. Additionally, incorporating more input parameters and increasing the dataset could further improve accuracy, making ML techniques a valuable tool in civil engineering.

Asadi et al. [97] explored the prediction of asphalt binder elastic recovery (ER) from Multiple Stress Creep Recovery (MSCR) [98] test results using ensemble learning methods. The ensemble models tested included tree-based bagging (RF, Extra Trees) and boosting methods (XGBoost, LightGBM, CatBoost). Extra Trees and XGBoost emerged as the most accurate models, demonstrating superior performance with R^2^ values of 0.852 and 0.842, respectively. These models surpassed traditional ER-DSR tests in predicting ER from MSCR results, despite differing temperature ranges. Key influential features identified were recovery at stress levels of 0.1 and 3.2 kPa. Clustering analysis revealed challenges in distinguishing patterns within the binders, suggesting potential improvements in MSCR analysis. Overall, the study advocates for the adoption of MSCR specifications over PG-Plus in asphalt binder characterization.

Shen et al. [99] developed ML models to predict the punching shear strength of FRP-reinforced concrete slabs, using a dataset of 121 experimental results. Several ML algorithms, including artificial neural network (ANN), SVM, decision tree (DT), and AdaBoost, were compared. AdaBoost demonstrated the best predicted accuracy with an RMSE of 29.83, MAE of 23.00, and R^2^ of 0.99. The empirical models and design codes were also compared, with GB 50010-2010 (2015) showing the best performance among the traditional models. SHAP [100] was used to interpret AdaBoost’s predictions, revealing the importance of variables such as the slab’s effective depth and the Young’s modulus of FRP reinforcement. The study highlighted that input variables, including the slab’s depth, significantly influence punching shear strength predictions. Overall, AdaBoost outperformed traditional models, making it a reliable tool for predicting shear strength in FRP-reinforced concrete slabs.

Rahman et al. [101] presented an extensive database and ML models to predict the shear capacity of reinforced concrete (RC) beams strengthened with FRP. The database includes 584 experimental results for rectangular and T-beams with 12 input features covering variations in beam geometry and FRP properties. Ten ML models, including CatBoost (CatB), XGBoost, and RF, were developed and validated using 10-fold cross-validation. CatB and XGB exhibited superior performance, achieving R^2^ values close to 0.9 and mean absolute errors below 0.25 kN, outperforming prior empirical models and design guidelines. SHAP identified the height of FRP layers and beam depth as key factors, while the type of fiber had minimal impact. The study emphasizes the need for updated databases to improve ML models and highlights the superior accuracy of ensemble learning techniques in predicting shear strength.

Table 3 presents an overview of studies employing ML techniques to predict polymer material properties and behavior across different applications. Each study utilizes a specific boosting algorithm to model and predict key properties, with an emphasis on model performance, key influencing factors, and any additional analysis methods used. The studies encompass a diverse range of materials, from polymers and composites to concrete and wastewater treatment, demonstrating the versatility and effectiveness of ML models in predicting complex material properties.

### 3.4. Advanced Manufacturing and Processing

Biruk-Urban et al. [102] investigated the machinability of new GFRP composites, focusing on the impact of drilling parameters on cutting forces and delamination. Four GFRP materials, varying in fiber type (plain or twill woven) and weight fraction (wf) ratio, were tested using a carbide diamond-coated drill. A novel ink penetration method was introduced to assess delamination, proving effective for detecting both push-out and peel-up delamination, as well as fiber pullouts. ML models were used to simulate the relationship between drilling parameters and delamination, with the Gradient Boosting Regressor achieving the highest accuracy. Results showed that feed per tooth significantly influenced delamination and cutting force amplitude, with lower values of feed per tooth reducing both. Twill fiber materials with lower wf ratios exhibited lower cutting forces and delamination factors, highlighting their machinability. The study offers insights for optimizing drilling processes and proposes future research on material properties, drill geometries, and advanced delamination detection techniques.

Jalali et al. [103] investigated the impedance properties of multi-walled carbon nanotube (MWCNT)/polystyrene nanocomposites synthesized via microwave-assisted in situ polymerization, examining the impact of microwave power, exposure time, and frequency. The Taguchi method and ANOVA identified microwave power as the most significant factor influencing impedance. A predictive model with an R^2^ of 0.96 was developed, showing high accuracy in predicting impedance values. ML models, including Decision Tree, RF, XGBoost, CatBoost, and LightGBM, were applied to enhance prediction accuracy. RF and CatBoost outperformed the other models, achieving R^2^ values of 0.9880 and 0.9811 on testing data, respectively. The results indicate that higher microwave power and extended exposure time increase impedance due to enhanced polystyrene content. This study demonstrates the potential of ML methods for accurately predicting impedance and tailoring the design of MWCNT-based composites for electrical applications.

Ma et al. [104] proposed using the XGBoost ML algorithm to predict the axial compressive capacity of CFRP-confined CFST short columns. The dataset, consisting of 379 data points from literature and experiments, includes factors such as concrete, steel, and CFRP strengths, cross-sectional areas, and section shapes. Eight ML algorithms, including XGBoost, were tested, with XGBoost showing the best prediction performance, achieving an R^2^ value of 0.9719. Hyperparameter optimization further improved the XGBoost model, increasing R^2^ to 0.9850. The study also identifies the importance of features like the cross-sectional area of core concrete and steel tube in determining compressive capacity. The optimized XGBoost model was highly accurate in predicting the axial capacity, outperforming other models, such as RF and Gradient Boosting Decision Trees. These findings suggest that XGBoost is an effective tool for predicting the behavior of CFRP-confined CFST short columns under axial compression.

Lignin plays a vital role in substituting synthetic polymers and reducing energy consumption, but traditional wet chemical methods for determining lignin content are inefficient and environmentally harmful. In the study by Gao et al. [105], the lignin content of Chinese fir was predicted using Raman spectroscopy, similar to a previous method for poplar. The peak at 2895 cm^−1^ was identified as the optimal internal standard, and the XGBoost algorithm demonstrated the highest prediction accuracy. Transfer learning was applied to improve the model’s accuracy and robustness, leading to an efficient, environmentally friendly method for predicting lignin content. Comparisons of nine algorithms revealed that advanced Gradient Boosting Machines (GBM) outperformed classic ML algorithms. Although the XGBoost model achieved a high test R^2^ and low RMSE, transfer learning was used to overcome challenges related to chemical structure differences. Ultimately, a reliable lignin content prediction model for Chinese fir, achieving a test R^2^ of 0.93, was successfully developed using XGBoost or LightGBM. This approach offers significant potential for more accurate and sustainable lignin content analysis.

Donga et al. [106] proposed a novel system for evaluating the hydrophobicity of insulated material surfaces using image processing and decision tree methods. A mixed image segmentation method is introduced to handle challenges like non-controlled illumination and nonstandard surfaces. The system uses four new characteristic parameters to describe the images of each sample, with classification performed using a MultiBoost decision tree, combining AdaBoost and Bagging algorithms. The results show that MultiBoost outperforms AdaBoost in classification accuracy, reducing errors and demonstrating better robustness, especially with k-fold cross-validation. The system uses a Digital Signal Processor (DSP) platform for training and testing, making it suitable for real-time applications. The study also highlights the limitations of traditional segmentation methods and suggests that the proposed approach is more versatile for uneven lighting images, though not universally applicable. Future work will focus on improving segmentation methods and developing adaptive algorithms for better accuracy in diverse conditions.

Kong [107] introduced an intelligent approach using hyperparameter optimization to predict the interfacial bond strength between FRP and concrete. By selecting CatBoost as the primary ML model, it outperformed eight other models, achieving an R_2_ of 0.9394 and MAPE of 1.21%. The hyperparameter optimization significantly improved the model’s accuracy, reducing dispersion by 90%. The optimized CatBoost model showed better performance than existing models in terms of R^2^, root mean square error, and coefficient of variation. The study also demonstrated that ML models, particularly the optimized CatBoost model, outperformed traditional bond strength models by 16.5% in mean accuracy and 14.19% in R^2^. Furthermore, grid searching was used to optimize the hyperparameters of models like CBR, MLP, and LightGBM, leading to enhanced prediction performance. The findings highlight the potential of hyperparameter optimization to improve the accuracy of predicting FRP-concrete bond strength, offering a reliable and efficient approach for future applications.

Membrane-based purification of therapeutic agents has gained significant attention as a promising alternative to traditional methods like distillation. Alanazi et al. [108] introduced a numerical approach employing multiple ML methods to predict solute concentration distributions during membrane-based separations. Key inputs, *r* and *z*, and a single target, *C*, were analyzed using over 8000 data points. Adaboost was applied to three base learners: k-nearest neighbors (KNN), linear regression (LR), and Gaussian process regression (GPR) [109]. The models were further optimized using the Bat Algorithm (BA). Boosted KNN achieved the highest R^2^ score of 0.9853, with low MAE and MAPE values, establishing it as the most accurate model. The boosted GPR model followed closely with robust predictive performance. These findings underscore the potential of ML-based strategies for improving membrane separation processes by providing high accuracy and insightful predictions.

Table 4 summarizes several recent studies that demonstrate the use of boosting techniques, focusing on their application in advanced manufacturing and processing settings:

### 3.5. Sustainability, Environmental, and Structural Performance

Tahir et al. [110] focused on designing novel polymer donors for organic solar cells using ML. Mordred descriptors were calculated for 271 polymer donors to train four ML models, with the gradient boosting regressor achieving the highest $R^{2}=0.85$. A chemical library of polymer donors was generated using BRICS, and similarity analysis using RDKit revealed clusters with strong structure–performance relationships. The 30 donors with the highest predicted power conversion efficiency (PCE) ranging from 9.13% to 9.44% were identified. Synthetic accessibility scores showed most polymers to be easily synthesizable (SA<6). Structural changes minimally impacted PCE, emphasizing the robustness of the designed materials.

Jiang et al. [111] developed ML models to predict amorphization and chemical stability during the hot-melt extrusion (HME) process for amorphous solid dispersions (ASDs). Using a dataset of 760 formulations, the study found that ECFP-LightGBM and ECFP-XGBoost are the most accurate models for predicting amorphization (92.8% accuracy) and chemical stability (96.0% accuracy), respectively. Key factors such as barrel temperature, drug loading, excipient ratios, and the chemical structure of active pharmaceutical ingredients (APIs) [112] significantly affect the results. SHAP and information gain analyses reveal important API substructures, such as chlorine atoms and nitrogen-containing heterocycles, that influence amorphization and stability. The study’s ML models can reduce trial-and-error in ASD development by accurately predicting amorphization and chemical degradation, ultimately streamlining the product development process. Additionally, the findings highlight the critical processing parameters, such as extruder configuration and screw speed, which influence both the amorphization and stability of ASDs.

A method for real-time monitoring of polymer agglomeration in a fluidized bed reactor (FBR) was developed by Pang et al. [113] using voiceprint feature recognition based on acoustic emission detection. Acoustic signals from polymer collisions on the reactor walls are collected, and voiceprint features are extracted using Mel Frequency Cepstrum Coefficients (MFCC) [114] and Linear Prediction Cepstrum Coefficients (LPCC). An improved Adaboost algorithm is proposed to classify these features, incorporating cost factors and the Gini index to better handle unbalanced small samples and improve accuracy. Experimental results from a fluidized bed pilot plant demonstrate the method’s effectiveness. The modified Adaboost algorithm outperforms the original in terms of classification accuracy, particularly for detecting micro-agglomeration and severe-agglomeration states. Performance evaluation metrics like the F-score indicate a significant improvement in prediction efficiency and accuracy. The method offers strong potential for industrial applications, particularly in the polyethylene production process, by enhancing agglomeration fault detection.

Fiosina et al. [115] employed advanced ML models to simulate and reverse engineer polymerization processes, addressing the challenges of tailoring polymer properties. Using data from a kinetic Monte Carlo simulator, ML methods (e.g., RF, XGBoost, and CatBoost) predicted key outputs, such as monomer concentration, average molar masses, and molar mass distributions [116] (MMDs) with high accuracy (R^2^ > 0.96). Reverse engineering models also demonstrated good agreement with targeted MMDs despite a lower R_2_ of 0.68. Multi-target regression (MTR) models outperformed single-output approaches by capturing dependencies among outputs. Explainability techniques validated the importance of input variables, aligning with expert expectations. Ensemble-based methods, particularly decision tree models, excelled in accuracy and scalability, reducing training data needs without performance loss. These results enable efficient prediction of polymerization recipes and conditions, advancing ML applications in polymer engineering. Future work will extend these methods to multi-objective optimization and complex polymer microstructures.

Deshpande et al. [117] investigated the prediction of the specific wear rate of glass-filled PTFE composite using ML algorithms, analyzing experimental data from a pin-on-disc wear testing machine. Various operating parameters, such as applied load, sliding velocity, and sliding distance, were varied using an orthogonal array L25 for experimentation. The data were analyzed using linear regression (LR), GB, and RF, with R_2_ values of 0.91, 0.97, and 0.94, respectively, showing the highest R^2^ value for the GB model, indicating an almost perfect fit. Pearson’s correlation analysis revealed that sliding distance and applied load significantly impacted the wear rate, while sliding velocity had a weaker effect. The experimental results showed a minimal wear rate of 3.04186 × 10^−5^ mm^3^/Nm at a load of 150 N, sliding velocity of 2 m/s, and sliding distance of 5000 m. The highest recorded wear rate was 4.410698 × 10^−5^ mm^3^/Nm. The study highlights the effectiveness of ML models in predicting wear rates and the importance of optimizing operating parameters for improved material performance.

Huang et al. [118] explored the influence of various factors on the open circuit voltage (Voc) of ternary polymer solar cells (PSCs) with non-fullerene acceptors (NFAs) using ML algorithms, such as XGBoost, k-nearest neighbor (KNN), and RF. The analysis reveals that the doping concentration of the third component has the greatest impact on Voc, with an optimal HOMO and LUMO energy level of the third component around −5.7 eV and −3.6 eV, respectively. The molecular descriptors (MDs) and molecular fingerprints (MFs) of the third component, such as hydrogen bond strength and aromatic ring structure, also significantly affect Voc. XGBoost was found to be the most accurate model for predicting Voc, with a low RMSE of 0.031 and MAE of 0.022. The study also highlights that the third component’s composition, including four methyl groups and two carbonyl groups, maximizes Voc. These findings offer valuable insights for designing and optimizing materials to enhance Voc in ternary PSCs, potentially improving their efficiency.

Plain concrete’s low tensile strain capacity (TSC) limits its performance, prompting the development of engineered cementitious composites (ECC) with polymer fibers to improve ductility. Inqiad et al. [119] aimed to predict ECC’s TSC using ML techniques, including Multi-Expression Programming (MEP), Gene Expression Programming (GEP), AdaBoost, and XGBoost. Among these, XGB achieved the highest accuracy, with a correlation coefficient of 0.986 and the lowest objective function (OF) value of 0.081. Shapley additive analysis revealed that fiber content, age, and water-to-binder ratio significantly impact TSC. While MEP and GEP provided empirical equations, XGB outperformed in precision. The study emphasizes the need for larger datasets and consideration of additional parameters like aggregate fineness and fiber properties to enhance model robustness and utility for predicting other ECC properties. These advancements support faster, cost-effective, and accurate ECC material evaluations.

Nguyen et al. [120] explored the flexural behavior of reinforced concrete beams using experimental tests and advanced ML models. Eight beams, incorporating varying proportions of recycled aggregates, fly ash, silica fume, and CFRP, were tested to analyze structural performance. A comprehensive dataset of 4851 samples enabled the application of ML frameworks, including RF, XGBoost, and LightGBM (LGBM), with hyperparameter tuning via Pareto optimization. Among the models, RFR demonstrated the highest accuracy, achieving the lowest MSE and effectively predicting flexural strength. Sensitivity analysis identified the key factors influencing beam performance, such as aggregate proportions, CS, and CFRP presence. Experimental results revealed notable improvements in CS (up to 53%) and load-bearing capacity (7%) for beams with recycled aggregates and silica fume. This study highlights the synergy of experimental analysis and ML techniques, advancing sustainable construction practices and optimizing structural design.

Table 5 summarizes sustainabilty studies, including targeted variables, the materials or properties predicted, the datasets and performance metrics, as well as the key factors influencing model predictions and additional techniques employed.

## 4. Review Outlook

**Figure 5:**
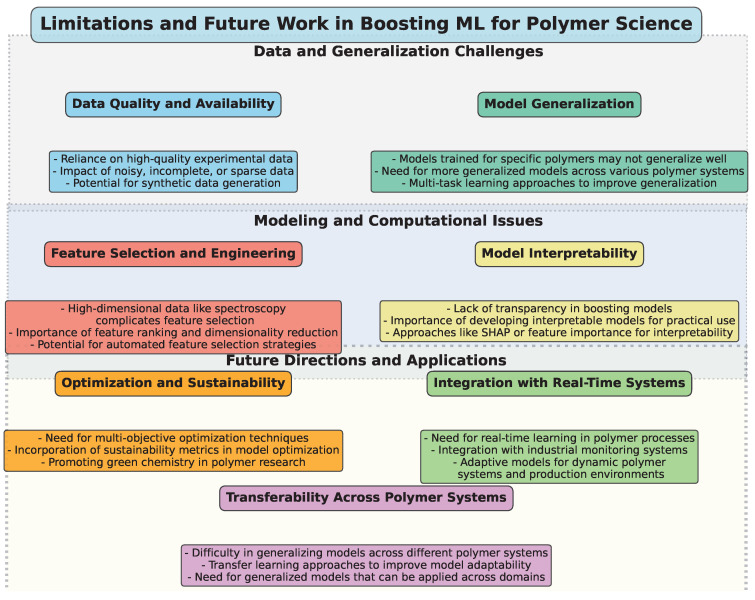
Illustrative diagram summarizing the limitations and future directions in the application of boosting ensemble learning in polymer science.

To provide a structured overview of the challenges, limitations, and potential future directions for the application of boosting methods in polymer science, a diagram (Figure 5) has been constructed. This visual representation organizes the discussed aspects into thematic groups, emphasizing their interrelations and collective impact on the field.

Boosting methods based on ensemble learning, such as Gradient Boosting and XGBoost, are being applied to capture the complex, non-linear relationships between polymer properties, processing parameters, and performance outcomes. Traditional techniques like linear regression and support vector machines are limited in modeling such interactions, whereas boosting methods iteratively combine multiple weak learners to form a comprehensive model [121]. This approach allows the model to learn intricate patterns within high-dimensional datasets without relying on extensive feature engineering, while reducing bias and variance compared to conventional techniques [122]. In addition, boosting methods integrate diverse input variables—including material characteristics, processing conditions, and environmental factors—into a single predictive framework [123]. Feature importance analysis within these models enables the identification of input variables that contribute most to the prediction, thus providing a clear basis for process optimization and material design in polymer science [124].

### 4.1. Analysis

In analyzing the boosting-based ML techniques applied to material property prediction in polymer science, the studies can be grouped into several thematic categories based on their application areas and focus.

The first group includes studies focused on predicting the mechanical properties of composites, such as CS, tensile strength, and load-bearing capacities of composites like short FRP composites, geopolymer concrete, and fly ash-based concrete. Boosting algorithms like XGBoost, LightGBM, and AdaBoost have consistently outperformed traditional models (e.g., decision trees or linear regression) in terms of prediction accuracy, as evidenced in studies like those by Zhao et al. [41] and Wang et al. [47]. The inclusion of feature importance analysis (e.g., SHAP) has enhanced the interpretability of results, helping to identify key factors influencing mechanical properties. However, despite high accuracy, some studies noted challenges in improving model reliability with sparse or incomplete datasets. For example, Katlav et al. [46] highlighted that expanding datasets and exploring advanced AI techniques could improve model performance. Additionally, data imbalance (e.g., in experimental datasets) remains an issue, which was addressed in some studies via techniques like SMOTE. Future work could focus on integrating multi-source data (e.g., experimental, simulation, and real-world operational data) to improve dataset diversity. Enhancing the models with deep learning techniques or hybrid models that combine boosting with neural networks might improve predictive accuracy and generalization. Furthermore, increasing the size and variety of experimental datasets could further reduce errors and improve the robustness of predictions.

The second group of studies focuses on sustainability and environmental impact prediction, particularly targeting carbon footprint predictions or the sustainable optimization of concrete materials. Techniques like adaptive boosting (AdaBoost-DTR) demonstrated excellent performance in predicting CO^2^ emissions in geopolymer concrete (Wudil et al. [55]). These models help optimize material formulations to reduce carbon emissions while maintaining structural integrity. The primary limitation in this category is the limited generalizability of models, as noted by Wudil et al. [55], due to the dataset’s focus on a specific type of geopolymer concrete and its limited size. There are also challenges related to incorporating more complex environmental data, such as lifecycle analysis or real-time IoT data. Broader datasets that incorporate different types of geopolymer concretes and alternative materials could improve model generalization. Additionally, combining boosting models with reinforcement learning or real-time monitoring systems could provide a more dynamic and accurate assessment of environmental impact in real-world applications.

The third group of studies focuses on geopolymer concrete and other sustainable materials. Ensemble methods like AdaBoost and RF Regression (RF) have been successfully applied to predict CS and other properties of geopolymer concrete (e.g., by Amin et al. [50]). These models have proven to be faster and more cost-effective than traditional experimental methods. However, a key limitation in this area is the inconsistent quality and range of datasets used for model training. For example, studies like those by Khan et al. [48] and Amin et al. [50] pointed out that the datasets were limited in terms of geographical diversity and specific material types. Expanding the dataset to include a wider range of geopolymer concrete formulations and real-world conditions (e.g., various curing times, environmental factors) could improve the accuracy and robustness of the predictions. Additionally, applying transfer learning or synthetic data generation techniques could help overcome dataset limitations by simulating material behavior in untested conditions.

XGBoost, LightGBM, and AdaBoost have shown promise in predicting complex material properties with high accuracy. These models are beneficial in the context of composite and sustainable materials, where traditional testing methods are time-consuming and costly. The ability to interpret feature importance (via SHAP or similar methods) is also a major advantage, allowing researchers to identify key factors influencing material performance. However, common limitations across studies include dataset quality (e.g., sparsity, imbalance, and lack of diversity), model overfitting, and generalization issues. Despite the promise of boosting techniques, the accuracy of predictions often depends on the quantity and quality of the data. Furthermore, computational costs and time constraints can be a challenge, especially in high-dimensional datasets or when incorporating complex simulations.

While CatBoost and LightGBM models offer high performance in handling structured data, their suitability varies depending on the specific characteristics of the dataset and prediction requirements. CatBoost is advantageous when dealing with categorical data, as it employs an ordered target-encoding method that prevents data leakage and overfitting [125]. This makes it well suited for scenarios where polymer formulations include categorical variables, such as material types, additive classifications, or process categories. LightGBM, on the other hand, relies on one-hot encoding or label encoding for categorical variables, which can sometimes lead to information loss or increased model complexity [126]. LightGBM is optimized for speed and memory efficiency, making it ideal for large datasets with high-dimensional numerical features [127]. Its histogram-based approach and leaf-wise tree growth strategy allow it to train faster and scale effectively, which is beneficial when modeling large experimental datasets involving high-resolution mechanical testing parameters, such as tensile strength, compression, shear, and fatigue resistance.

For progressive predictions involving dynamic process parameters—such as temperature, pressure, additives, and time variations in polymer molding—LightGBM can be more efficient due to its ability to handle numerical features and complex interactions with reduced computation time [128,129]. However, CatBoost can provide more stable and interpretable predictions when categorical variables significantly influence the mechanical properties of polymer materials [130,131,132]. CatBoost has built-in Bayesian bootstrapping and an ordered boosting method that improve its robustness to noisy or imbalanced datasets, making it suitable when working with experimental data that may have missing values or skewed distributions [133]. LightGBM, while also capable of handling imbalanced data, may require additional techniques, such as balanced weight adjustments or custom loss functions, to achieve comparable performance [134].

### 4.2. Limitations

One of the primary limitations is the quality and availability of data. Many studies, such as those predicting lignin content [105] and polymer agglomeration [113], relied on relatively small or unbalanced datasets, which can constrain the reliability and generalizability of ML models. To enhance model performance, future research could focus on expanding and diversifying datasets, potentially through automated data collection methods or collaborative efforts within the research community.

Another challenge is the interpretability of boosting models. While these models often achieve high accuracy, their complexity can make them difficult to understand, which limits their adoption in practical applications. For instance, in studies like [111,119], explainability techniques such as SHAP were employed to gain insights into model behavior, but these techniques need to be further developed to provide clear, actionable insights for stakeholders. Ensuring that models are both accurate and interpretable will be crucial for their widespread acceptance, especially in industries where decision-makers require transparent models to trust automated predictions.

The process of feature selection and engineering also impacts in the success of boosting models. Studies such as [118] demonstrate the importance of molecular descriptors for predicting polymer properties, but automating and improving feature selection processes remains a challenge. Future work could focus on developing more advanced algorithms for feature engineering or domain-specific heuristics that would reduce the burden on researchers and improve model performance.

Moreover, while transfer learning was successfully applied in some studies, such as in the prediction of lignin content [105], generalizing across different polymer systems remains difficult. The need for transferability across polymer systems is essential for building ML models that are universally applicable. Future research could explore the development of generalized frameworks or domain-adaptation techniques that would reduce the dependency on system-specific training data, enhancing the flexibility and scalability of boosting models across a broader range of polymer materials.

Furthermore, although boosting methods have proven effective for predicting outcomes, their integration into experimental workflows has been somewhat limited. For example, the real-time monitoring of polymer agglomeration [113] and the reverse engineering of polymerization processes [115] show great promise, but their validation in industrial settings remains an ongoing challenge. In the future, more robust hybrid frameworks that combine ML models with real-time monitoring systems will be needed to accelerate the adoption of these methods in practical applications.

### 4.3. Future Work

Another important area for future work is optimization beyond prediction. Most of the studies reviewed emphasized predictive accuracy, but few addressed the optimization of polymer properties, synthesis conditions, or structural designs. A promising direction for future research is the application of multi-objective optimization techniques to balance trade-offs between factors such as performance, cost, and sustainability, as suggested in [115]. This approach could lead to better-designed materials and processes that fulfill the demands of both industry and sustainability.

Sustainability and the role of green chemistry in polymer science are also crucial considerations. Several studies, such as those predicting lignin content [105] and designing polymer donors for organic solar cells [110], emphasized the potential for ML to guide the development of eco-friendly materials. However, future research should expand on this by incorporating life cycle assessments and environmental impact metrics into ML models. This would help ensure that the polymers being designed not only perform well but also minimize their ecological footprint.

Finally, real-time learning systems are an essential future direction. Polymer processes, such as those in fluidized bed reactors [113] or wear testing [117], are highly dynamic, and the ability to adapt to evolving data streams is critical. Future research should focus on developing online learning algorithms capable of maintaining the efficiency of boosting methods in such dynamic environments. These adaptive systems will be crucial for improving accuracy and efficiency in real-time polymer processing and product development.

Moving forward, integrating multiple ML paradigms (such as combining ensemble methods with deep learning) could enhance predictive performance. Additionally, the application of hybrid models that combine the strengths of simulation-based methods with ML could allow for better-informed predictions. For example, Ghasem [135] presented a Computational Fluid Dynamics (CFD) and AI/ML simulation of a polypropylene fluidized bed reactor, aiming to reduce reactor loss and enhance process understanding. By combining CFD with machine learning algorithms, the simulation accurately predicts reactor performance and identifies key operating parameters to optimize polypropylene yield and reactor efficiency. Ensuring that datasets are diverse, well curated, and representative of real-world scenarios will be key to improving model reliability and performance. By addressing the limitations related to data quality, model complexity, and computational efficiency, boosting-based ML models could offer substantial improvements in predicting and optimizing material properties, accelerating the development of advanced polymer and composite materials.

In conclusion, while boosting-based ML methods have shown considerable promise in addressing the challenges of polymer science, future work must tackle the limitations outlined above. By focusing on improving data quality, model interpretability, feature engineering, transferability, and real-time adaptation, as well as integrating optimization and sustainability considerations, boosting methods can become indispensable tools for advancing both fundamental research and industrial applications in polymer science.

## Figures and Tables

**Table 1:** Comparison of results for studies related to concrete and GPCs.

Study	Boosting Technique	Application	Materials/ Properties Predicted	Dataset	Model Performance (R^2^, MAE, RMSE)	Key Influencing Factors	Additional Techniques/ Analysis
Zhao et al. (2023) [41]	XGBoost, LightGBM, Extra Trees	Short FRP composites	Homogenized mechanical properties (e.g., Young’s modulus)	High fidelity composite datasets, experimental data	R^2^ of 0.988 (train), 0.952 (test)	Fiber orientation, fiber content, matrix Young’s modulus	SHAP analysis, micromechanical model integration
Zhang et al. (2023) [44]	CatBoost, RF, Ridge, LASSO	Steel-reinforced concrete columns (SRCCs) clad in CFRP	Axial compression load capacity	Sparse data, 12 features	High predictive accuracy, better than individual models	Load capacity factors	SMOTE for data balancing, SHAP analysis
Katlav et al. (2024) [46]	XGBoost, LightGBM, AdaBoost, RF	UHPGC	Compressive strength (CS)	181 test results, 13 input features	R^2^ = 0.948	Age, fiber content, water content	SHAP analysis, user interface for practical predictions
Wang et al. (2022) [47]	AdaBoost, RF	GPCs	CS	Experimental datasets	R^2^ = 0.90	Fly ash, curing time, NaOH molarity	SHAP analysis
Khan et al. (2022) [48]	XGBoost, GB	GPCs	CS	500+ mixes	R^2^ = 0.98	GGBS, NaOH molarity, fly ash	SHAP analysis
Zhou et al. (2024) [49]	XGBoost, AdaBoost, Gradient Boosting	GPC	CS, STS	Experimental data	R^2^ > 0.90	Blast furnace slag, curing duration, fine aggregate	K-fold analysis
Amin et al. (2022) [50]	AdaBoost, RF	GeoPC	CS	481 mixes, 9 variables	R^2^ = 0.95	Curing time, temperature, specimen age	Sensitivity analysis, k-fold validation
Ansari et al. (2023) [52]	AdaBoost	GPC with fly ash	CS	154 datasets	R^2^ = 0.944, RMSE = 2.506, MAE = 1.259	Fly ash content, water-to-binder ratio	Evaluation through R^2^, MAE, RMSE
Dodo et al. (2024) [53]	AdaBoost, Bagging with ANN	FASBGeoPC	CS	156 data points	R^2^ = 0.914	GGBS, NaOH molarity, temperature	SHAP analysis, ensemble methods
Wudil et al. (2025) [55]	AdaBoost	Fly ash GeoPC	Carbon dioxide footprint (CO_2_-FP)	Experimental data, material features	CC = 0.9665, NSE = 0.9343	NaOH, curing temperature, fly ash content	SHAP analysis, IoT integration

**Table 2:** Summary of studies applying ensemble boosting techniques in predicting properties of FRP and reinforced concrete systems.

Study	Boosting Technique	Application	Materials/ Properties Predicted	Dataset	Model Performance (R^2^, MAE, RMSE)	Key Influencing Factors	Additional Techniques/ Analysis
Kim et al. [56]	CatBoost	FRP-concrete bond strength	FRP bond strength	855 shear test data	RMSE: 2.31, R^2^: 0.96	Small dataset, categorical features	Compared with XGBoost, HGBoost, RF
Kumarawadu et al. [57]	XGBoost, CatBoost	Fire resistance of FRP-strengthened RC beams	Fire resistance	21,000 data points	Accuracy: >92%	Loading ratio, insulation depth, concrete cover	Bayesian optimization, SHAP analysis
Wang et al. [58]	XGBoost	Shear strength of FRP-RC beams	Shear strength	442 RC beam data	High prediction accuracy	Effective height of FRP, shear span ratio	Isolation forest anomaly detection
Mahmoudian et al. [59]	Decision Tree, RF, AdaBoost, XGBoost	Flexural bond strength of GFRP	GFRP-concrete bond	Experimental data	Accuracy: 100%	Concrete type, GFRP bar properties	Hyperparameter tuning, SHAP analysis
Mahmoudian et al. [60]	AdaBoost, XGBoost, CatBoost, GB, Hist GB	Bond strength in FRP-UHPC	FRP-UHPC bond strength	Experimental dataset	R^2^: 0.95, RMSE: 2.21	Tensile strength, elastic modulus, embedment length	Shapley values, Voting Regressor
Wang et al. [61]	LightGBM, Genetic Programming	Fire resistance of FRP-strengthened RC beams	Fire resistance, deflection	20,000 data points	R^2^: 0.923 (Fire Resistance), 0.789 (Deflection)	Insulation thickness, reinforcement area	Genetic Algorithm, SHAP analysis
Hu et al. [62]	XGBoost, Gradient Boosting	CFRP/metal composite laminates’ mechanical properties	Tensile and bending strength	Experimental and simulation data	Best for tensile (XGBoost), bending (RF)	Laminate stacking sequence	Numerical and experimental integration
Aydın et al. [63]	DMLP, RF, GBR, LR, PR	Wear behavior of MWCNT-CFRP composites	Wear loss prediction	Experimental data	R^2^: 0.9726	MWCNT content, load, sliding distance	SEM, EDS analysis
Li et al. [64]	RF, AdaBoost	Bond strength of BFRP-concrete in corrosive environments	BFRP-concrete bond strength	355 samples	R^2^: 0.925, MAE: 0.0589	Corrosion, concrete strength, BFRP properties	SHAP analysis
Khodadadi et al. [65]	PSO-CatBoost	Compressive strength of CFRP-confined concrete	CFRP-CC compressive strength	916 experimental results	R^2^: 0.9572	CFRP reinforcement ratio, unconfined CS	SHAP, PFI, Graphical interface
Alizamir et al. [68]	GBRT, RF, ANNMLP, ANNRBF	FRP-confinement in concrete strength	Concrete strength ratio	765 specimens	RMSE reduction: 69.94% (GBRT)	Concrete type, specimen geometry	Advanced feature selection
Amin et al. [50]	DT, GBT	Flexural capacity of FRP-RC beams	Flexural strength	60% training, 40% validation	R: 0.94 (GBT)	Beam depth, concrete CS	Sensitivity analysis
Amin et al. [70]	RF, XGBoost, LIGHT GBM	Bond strength of FRP on concrete prisms	Interfacial bond strength (IBS)	70% training, 30% testing	R^2^: 0.942 (training), 0.865 (testing)	FRP thickness, elastic modulus	SHAP analysis
Tian et al. [71]	CatBoost	Bond strength of FRP bars to concrete	Bond strength	158 pull-out test results	RMSE reduction: 58.3%	Rib spacing and width, concrete properties	Integration with traditional formulas

**Table 3:** Summary of studies applying boosting techniques in polymer materials properties prediction.

Study	Boosting Technique	Application	Materials/ Properties Predicted	Dataset	Model Performance (R^2^, MAE, RMSE)	Key Influencing Factors	Additional Techniques/ Analysis
Cheng et al. [72]	XGBoost, LightGBM, CatBoost	Friction coefficient of polymer–metal pairs	Friction coefficient, temperature range (−120 °C to 25 °C)	Various working conditions	RMSE: 0.0135, R^2^: 0.615	Friction noise, temperature	Time-frequency feature analysis
Fatriansyah et al. [73]	XGBoost, ANN, RNN, KNN, SVR	Glass transition temperature (Tg) of polymers	Tg of polymers	SMILES descriptors	R^2^: 0.774, MAE: 9.76% deviation	SMILES descriptor length	One Hot Encoding vs NLP
Ascencio-Medina et al. [74]	GBR	Dielectric permittivity of polymers	Dielectric permittivity	86 polymers	R^2^: 0.938 (train), 0.822 (test)	Electronic, ionic, dipolar polarization	Genetic algorithm, ALE analysis
Goh et al. [78]	LightGBM (LGB-Stack)	Polymer properties prediction	Various polymer properties	4209 polymers	R^2^: 0.92, RMSE: 0.41	Molecular fingerprints	Feature reduction, Recursive Feature Elimination
Rajaee et al. [80]	AdaBoost, Decision Tree	Mechanical	Tensile strength, Young’s modulus, elongation	Polypropylene nanocomposites	R^2^: 0.90 for Young’s modulus	TPO levels, nanoparticle content	Sensitivity analysis
Abdi et al. [82]	CatBoost	Photodegradation of tetracycline	TC degradation from wastewater	374 data points	AAPRE: 1.19%, STD: 0.0431	Catalyst dosage, pH, surface area	Outlier detection
Okada et al. [85]	GBM-RFE	Hydrophilicity of polymer coatings	Surface hydrophilicity	Polyacrylamide coatings	High accuracy in feature selection	Polymer chain dynamics	TD-NMR, Recursive Feature Elimination
Salehi et al. [86]	CatBoost, XGBoost, LightGBM, RF	Rheological properties of RPMB	Complex shear modulus, phase angle	Recycled plastic modified bitumen	R^2^: 0.98 (shear modulus)	Base bitumen, recycled plastic quantity	SHAP analysis
Chonghyo et al. [89]	CatBoost, XGBoost, MLR	Heat deflection temperature (HDT) of PPCs	Heat deflection temperature	Polypropylene composites	R^2^: 0.8965, RMSE: 7.3477	Material composition	Novel dimensionless number “A”
Chepurnenko et al. [90]	CatBoost, Evolutionary algorithms	Rheological properties of polymers	Viscosity, velocity modulus	Epoxy binder	MAPE: 0.86, MSE: 0.001	Stress relaxation	Data normalization, regularization
Hofmann et al. [91]	LightGBM	Local solidity in PBF-LB process	Porosity, solidity	Thermal and temporal features	High prediction accuracy	Peak temperature, reheating	Infrared thermography, X-ray micro-CT
Gadagi et al. [92]	XGBoost, AdaBoost, GBM	Surface roughness of composites	Surface roughness of epoxy composites	Jute/basalt composites	High accuracy in roughness prediction	Spindle speed, feed rate	Taguchi L27 array
Wang et al. [93]	ICA-LightGBM	Geo-polymer concrete CS prediction	Compressive strength (CS) of geo-polymer concrete	Geo-polymer concrete dataset	R^2^: 0.9871 (train), 0.9805 (test)	Hyperparameter optimization	Imperialist Competitive Algorithm optimization
Ahmad et al. [96]	Boosting, AdaBoost	Compressive strength of GPC	Compressive strength of GPC	High calcium fly-ash-based GPC	R^2^: 0.96	Fly ash composition	Sensitivity analysis
Asadi et al. [97]	XGBoost, LightGBM, CatBoost, Extra Trees	Asphalt binder elastic recovery (ER) prediction	Elastic recovery (ER) from MSCR test results	Asphalt binders	R^2^: 0.852 (Extra Trees), 0.842 (XGBoost)	Stress recovery at 0.1, 3.2 kPa	Clustering analysis
Shen et al. [99]	AdaBoost	Punching shear strength of FRP RC slabs	Punching shear strength of FRP RC slabs	121 experimental results	R^2^: 0.99, RMSE: 29.83, MAE: 23.00	Effective depth, Young’s modulus of FRP	SHAP analysis
Rahman et al. [101]	CatBoost, XGBoost	Shear capacity of FRP RC beams	Shear capacity of FRP RC beams	584 experimental results	R^2^: 0.9, MAE: 0.25 kN	FRP layer height, beam depth	SHAP analysis

**Table 4:** Summary related to polymers advanced manufacturing and processing studies.

Study	Boosting Technique	Application	Materials/ Properties Predicted	Dataset	Model Performance (R^2^, MAE, RMSE)	Key Influencing Factors	Additional Techniques/ Analysis
Biruk-Urban et al. [102]	GB	GFRP composites machinability	Cutting forces, delamination	Carbide diamond-coated drill data	High accuracy in delamination prediction	Drilling parameters, fiber type, weight fraction	Novel ink penetration method for delamination detection
Jalali et al. [103]	RF, CatBoost	MWCNT-polystyrene nanocomposites impedance	Impedance properties	Microwave-assisted synthesis data	R^2^ = 0.9880 (RF)	Microwave power, exposure time, frequency	Taguchi method, ANOVA for feature importance
Ma et al. [104]	XGBoost	CFRP-confined CFST short columns	Axial compressive capacity	379 data points from literature	R^2^ = 0.9850 after hyperparameter optimization	Concrete, steel, CFRP strengths, cross-sectional area	Hyperparameter optimization for improved accuracy
Gao et al. [105]	XGBoost, LightGBM	Lignin content prediction in Chinese fir	Lignin content	Raman spectroscopy data	R^2^ = 0.93 (XGBoost)	Raman peaks, chemical structure differences	Transfer learning for model improvement
Donga et al. [106]	MultiBoost (AdaBoost + Bagging)	Hydrophobicity evaluation of insulated materials	Hydrophobicity properties	Image data from surface samples	High classification accuracy with MultiBoost	Illumination and surface irregularities	Image segmentation, DSP platform for real-time training
Kong [107]	CatBoost	FRP-concrete bond strength prediction	Bond strength	Experimental data	R^2^ = 0.9394, MAPE = 1.21%	Interfacial bond strength	Hyperparameter optimization, grid search
Alanazi et al. [108]	Adaboost	Membrane separation process in therapeutic agent purification	Solute concentration distribution	Over 8000 data points from experiments	R^2^ = 0.9853 (Boosted KNN)	Solute concentration, membrane parameters	Bat Algorithm for model optimization

**Table 5:** Summary of polymers studies on sustainability, environmental, and structural performance.

Study	Boosting Technique	Application	Materials/ Properties Predicted	Dataset	Model Performance (R^2^, MAE, RMSE)	Key Influencing Factors	Additional Techniques/ Analysis
Gao et al. [105]	XGBoost, LightGBM	Lignin content prediction	Lignin content in Chinese fir	Raman spectroscopy data	Test R^2^ = 0.93	Raman peak (2895 cm^−1^), chemical structure differences	Transfer learning; comparison of 9 algorithms
Tahir et al. [110]	Gradient Boosting Regressor	Design of polymer donors for OSCs	Predicted power conversion efficiency (PCE)	Mordred descriptors for 271 polymer donors	$R^{2}=0.85$	Molecular structure, synthetic accessibility	BRICS-based chemical library; RDKit similarity analysis
Jiang et al. [111]	ECFP-LightGBM, ECFP-XGBoost	Hot-melt extrusion for ASDs	Amorphization and chemical stability	760 formulation data points	Accuracy: 92.8% (amorphization), 96.0% (stability)	Barrel temperature, drug loading, API substructures	SHAP and information gain analyses
Pang et al. [113]	Improved AdaBoost	Real-time monitoring in FBR	Polymer agglomeration states	Acoustic emission signals (MFCC, LPCC)	Improved classification accuracy (F-score elevated)	Acoustic features affected by illumination	Cost factors and Gini index integration; DSP platform
Fiosina et al. [115]	XGBoost, CatBoost	Reverse engineering polymerization	Monomer concentration, molar masses, MMDs	Kinetic Monte Carlo simulator data	R^2^ > 0.96 for predictions; 0.68 for reverse engineering	Polymerization kinetics input variables	Multi-target regression; explainability techniques
Deshpande et al. [117]	Gradient Boosting (GB)	Wear rate prediction in composites	Specific wear rate of glass-filled PTFE	Pin-on-disc wear test data (L25 array)	R^2^ = 0.97 (GB model)	Sliding distance, applied load, sliding velocity	Pearson’s correlation analysis
Huang et al. [118]	XGBoost	OSC performance optimization	Open circuit voltage (Voc) of ternary PSCs	Data on polymer solar cells with NFAs	RMSE = 0.031, MAE = 0.022	Doping concentration, HOMO/LUMO levels, MDs	Molecular descriptor and fingerprint analysis
Inqiad et al. [119]	XGBoost	ECC TSC prediction	TSC of ECC	Experimental ECC data	Correlation coefficient = 0.986, OF = 0.081	Fiber content, age, water-to-binder ratio	Comparison with MEP and GEP; Shapley additive analysis
Nguyen et al. [120]	XGBoost, LightGBM, RF	Flexural behavior of RC beams	Flexural strength	4851 experimental samples	RF achieved lowest MSE (highest accuracy)	Aggregate proportions, compressive strength, CFRP presence	Pareto optimization for hyperparameter tuning; sensitivity analysis
